# Holistic Molecular Design of Ionic Surfaces for Tailored Water Wettability and Technical Applications

**DOI:** 10.3390/nano15080591

**Published:** 2025-04-11

**Authors:** Huiyun Wang, Chongling Cheng, Dayang Wang

**Affiliations:** 1 State Key Laboratory for Inorganic Synthesis and Preparative Chemistry, College of Chemistry, Jilin University, Changchun 130012, China; huiyun23@mails.jlu.edu.cn; 2 State Key Laboratory of Digital Medical Engineering, School of Biological Science and Medical Engineering, Southeast University, Nanjing 210096, China

**Keywords:** ionic surfaces, polyelectrolytes, hydrophilic coating, wetting, surface hydration

## Abstract

This comprehensive review systematically explores the molecular design and functional applications of nano-smooth hydrophilic ionic polymer surfaces. Beginning with advanced fabrication strategies—including plasma treatment, surface grafting, and layer-by-layer assembly—we critically evaluate their efficacy in eliminating surface irregularities and tailoring wettability. Central to this discussion are the types of ionic groups, molecular configurations, and counterion hydration effects, which collectively govern macroscopic hydrophilicity through electrostatic interactions, hydrogen bonding, and molecular reorganization. By bridging molecular-level insights with application-driven design, we highlight breakthroughs in oil–water separation, anti-fogging, anti-icing, and anti-waxing technologies, where precise control over ionic group density, the hydration layer’s stability, and the degree of perfection enable exceptional performance. Case studies demonstrate how zwitterionic architectures, pH-responsive coatings, and biomimetic interfaces address real-world challenges in industrial and biomedical settings. In conclusion, we synthesize the molecular mechanisms governing hydrophilic ionic surfaces and identify key research directions to address future material challenges. This review bridges critical gaps in the current understanding of molecular-level determinants of wettability while providing actionable design principles for engineered hydrophilic surfaces.

## 1. Introduction

**Figure 1:**
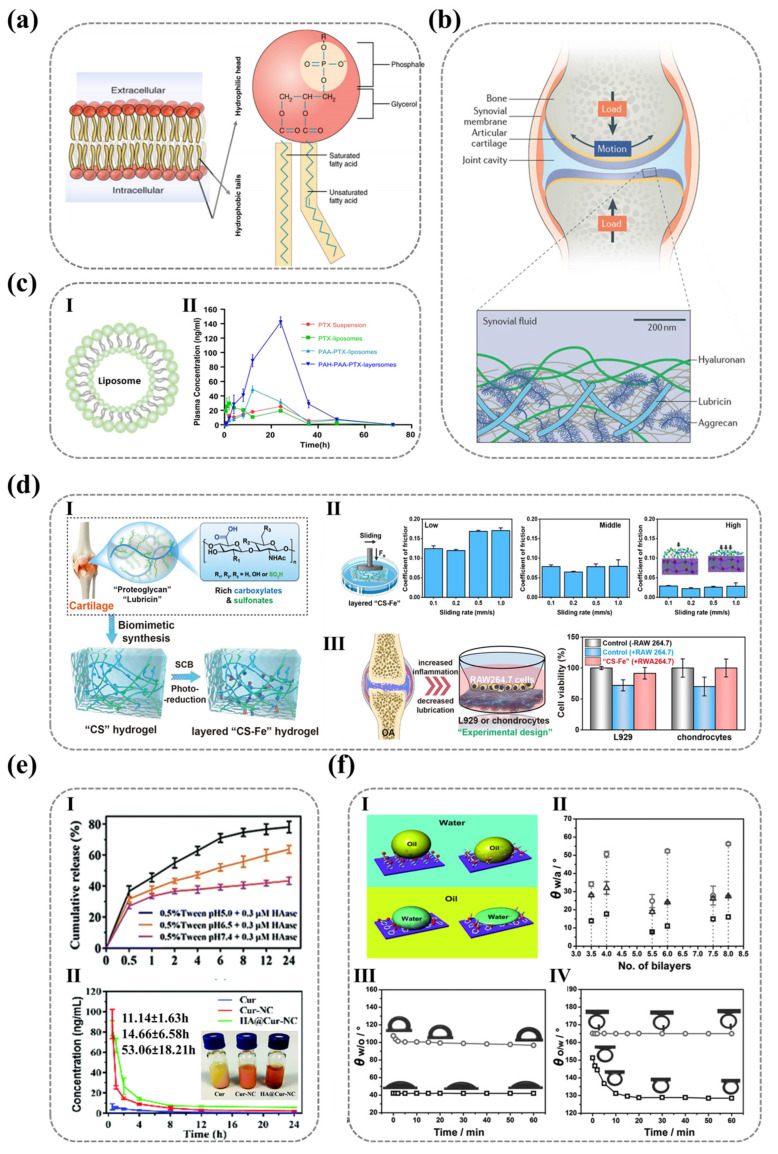
**The critical role of ionic functional groups in controlling surface wettability across diverse biomaterials**. (**a**) Phospholipid bilayers leverage ionic groups to form hydration layers, influencing surface interactions and wettability. Ref. [20] Copyright from *Elsevier*@2022. (**b**) Cartilage surfaces utilize charged macromolecules (e.g., hyaluronic acid, lubricin, and phospholipids [15]) to achieve superior hydration and lubrication under high pressure, showcasing the dynamic control of wettability. Ref. [21]. Copyright from *Wiley@2021.* (**c**) Polyelectrolyte-modified liposomes employ ionic groups to enhance drug delivery efficiency and biocompatibility, illustrating how surface chemistry regulates wettability for sustained release. I. Basic structure of liposomes. II.Plasma concentration-time curves of PTX suspension, PTX liposomes, PAA-PTX liposomes, and PAH-PAA-PTX layered formulations. Ref. [16]. Copyright from *Elsevier*@2012. (**d**) Hydrogels with abundant ionic groups (e.g., carboxylate/sulfonate) exhibit super-lubrication properties, mimicking natural cartilage and highlighting the role of ionic groups in modulating wettability and tribological performance. I. Schematic of anionic hydrogel “CS-Fe3+” rich in carboxylate/sulfonate. II. Schematic of tribological testing of layered “CS-Fe” hydrogel and the relationship between sliding velocity and friction coefficient (COF) under low, medium, and high load conditions. III. Experimental design simulating osteoarthritis (OA) and cell viability of L929 cells and chondrocytes co-cultured with RAW264.7 cells regulated by “CS-Fe” hydrogel. Ref. [17]. Copyright from *ACS*@2022. (**e**) Curcumin nanocomposites (Cur-NCs) demonstrate pH-dependent release and plasma concentration profiles, emphasizing how ionic groups on surfaces can be tailored to control wettability and drug delivery kinetics. I. Release behavior of Cur and Cur-NCs at different pH values. II. Appearance and average plasma concentration-time curves of Cur, Cur-NCs, and HA@Cur-NCs. Their respective blood elimination half-lives are also noted. Ref. [18]. Copyright from *RSC*@2019. (**f**) Polyelectrolyte multilayers (e.g., PDDA/PSS) exhibit distinct wettability behaviors in air and oil phases, revealing how the spatial arrangement of ionic groups influences surface interactions and wettability. I. Schematic of molecular properties and wetting behavior of PDDA and PSS in air and oil phases. II. Contact angle values of freshly prepared (square), 72-h aged at room temperature (triangle), and 72-h aged at 60 °C (circle) PEMs with varying bilayer numbers in air. III. and IV. Contact angle and water-in-oil contact angle curves for (PDDA/PSS)_3.5_ (square) and (PDDA/PSS)_4_ (circle) in oil over time. Ref. [19]. Copyright from *Wiley*@2015.

The molecular-scale integration of hydrophilic ionic motifs on solid surfaces has emerged as a cornerstone of advanced material design, where the synergistic action of polar functional groups and surface charge determines the interface wettability, hydration stability, and efficiency. These ionic architectures leverage synergistic hydrogen bonding and electrostatic interactions with water molecules to engineer surfaces with programmable hydrophilicity, enabling applications ranging from self-cleaning coatings to bioadhesive interfaces. Nature offers masterclasses in such molecular engineering: phospholipid bilayers employ densely packed ionic headgroups (e.g., phosphatidylcholine and phosphatidylserine) to construct ≈1 nm hydration layers that regulate ion channel permeability (Figure 1a) [1,2,3,4,5,6,7,8,9], while cartilage surfaces exploit ultra-dense carboxylate arrays to achieve superlubricity (friction coefficient ≈ 0.001) under megapascal pressures via hydration-mediated boundary lubrication (Figure 1b) [10,11,12,13,14,15]. These biological systems exemplify how precise control over interfacial hydration states—governed by ionic group density, charge distribution, and supramolecular assembly—can impart specialized functionalities through water-structuring phenomena.

Inspired by these natural paradigms, synthetic strategies have harnessed ionic surface engineering to transcend conventional material limitations [16,17,18,19]. Layer-by-layer polyelectrolyte assemblies on liposomes (PAA/PAH, Figure 1c) enhance drug bioavailability through charge-modulated hydration stability [16], while carboxylate/sulfonate-enriched hydrogels replicate cartilage-like tribological performance via ionic group density optimization (Figure 1d) [17]. Further innovations, such as pH-responsive hyaluronic acid coatings on nanocrystals (HA@Cur-NCs, Figure 1e), demonstrate how dynamic hydration layer restructuring enables tumor-targeted drug release with extended circulatory half-lives [18]. Yet, despite these advances, critical knowledge gaps persist in correlating molecular-scale ionic features—polarity, spatial configuration, and counterion mobility—with macroscopic wettability. Current models often oversimplify hydrophilicity as a static function of chemical group polarity or density, neglecting dynamic factors such as molecular reorientation under environmental influences and hydration efficiency related to the counter-ion coordination with the ionic polymer. For instance, polyelectrolyte multilayers (PDDA/PSS) with identical roughness (<3 nm) exhibit environmental polarity-dependent wettability divergences: Based on the contact angle measurements, the wettability of the PDDA surface shows minimal variation between nonpolar and polar phases, whereas the PSS surface exhibits significant wettability differences (Figure 1f) [19]. It can be observed that for nanoscopically smooth surfaces, wettability differences arise not only from material-specific hydrophilicity but also from other factors affecting the same hydrophilic groups. This anomaly highlights the limitations of conventional models in explaining surface hydrophilicity at the molecular scale.

To address these challenges, this review begins by examining conventional theoretical models, emphasizing the importance of deconstructing surface wettability at the molecular level and the suitability of ionic polymer surfaces as model systems. We then introduce current fabrication techniques for ionic polymer surfaces and establish a framework to compare their mechanical advantages and limitations. Furthermore, from a molecular perspective, we elucidate how ionic polymer surfaces modulate macroscopic hydrophilicity through microscopic control, focusing on the types of ionic groups, molecular configurations, and the hydration effects of counterions.

Moreover, building upon this foundation, the discussion pivots to application-driven design principles, where industrial and biomedical requirements—amphiphilic membrane systems for oil–water separation, stimuli-responsive anti-icing/anti-fogging interfaces, and bioinspired antifouling coatings—are translated into tailored molecular blueprints. Case studies illustrate how zwitterionic architectures, pH-triggered hydration layers, and biomimetic charge gradients enable breakthrough functionalities. Finally, we consolidate the molecular mechanisms underlying hydrophilic ionic polymer surfaces, and by bridging molecular-scale insights with macroscopic properties, we propose promising research directions to address future challenges in materials science.

## 2. Design Approaches for Hydrophilic Ionic Polymer Surfaces

Hydrophilicity is a fundamental characteristic that describes the ability of water to wet a solid surface, specifically its ability to replace another fluid on the surface. The degree to which water spreads on a surface is the most macroscopic manifestation of hydrophilicity. In the process of wetting, the study of hydrophilic surfaces is usually conducted in a three-phase system involving water, air, and a solid or water, oil, and a solid. When a liquid droplet contacts a surface, the smaller the angle between the tangent of the gas-liquid interface and the solid–liquid interface (i.e., the contact angle (θ)), the stronger the spreading ability of the water droplet, indicating better hydrophilicity of the surface. From a microscopic perspective, hydrophilicity describes the interaction ability between the solid surface material molecules and water molecules. Materials with high hydrophilicity can form strong adsorption interactions with water molecules.

**Figure 2:**
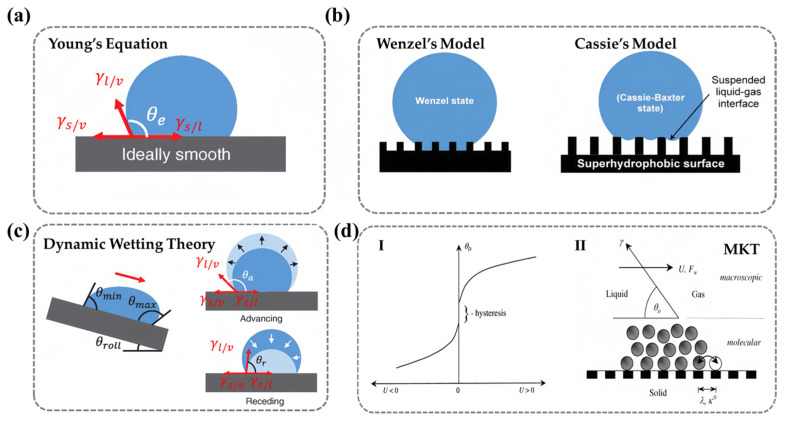
**Schematic diagram of wettability basic theory:** (**a**) Young’s Equation. (**b**) Wenzel’s Model (**Left**) and Cassie’s Model (**Right**) (single substance with air). (**c**) Dynamic wetting theory (the contact angle hysteresis, the advancing angle, and the receding angle). (**a**–**c**) Ref. [36] Copyright from *Wiley*. (**d**) Molecular kinetics theory. I. Velocity-dependence of the contact angle for a partially wetting liquid. II. Dynamic wetting according to the molecular-kinetic theory. Ref. [33] Copyright from *Elsevier*.

As early as the beginning of the 19th century, Thomas Young first related the macroscopic equilibrium contact angle ($\theta_{e}$) to the microscopic molecular interactions at the liquid/vapor/solid three-phase contact line. He derived a trigonometric equation between the contact angle and the interfacial tension, providing a quantitative analytical tool for understanding the interaction between liquids and solids (Figure 2a) [22]:

(1) $$\gamma_{l/v}\cos\theta_{e}=\gamma_{s/v}-\gamma_{s/l}$$

where $\gamma_{l/v}$ is the interfacial tension between liquid and vapor, $\gamma_{s/v}$ is the interfacial tension between the solid and vapor, and $\gamma_{s/l}$ is the interfacial tension between the solid and liquid. When $\gamma_{s/v}$ is greater than $\gamma_{s/l}$, liquid tends to spread across the surface to minimize system energy, leading to $\theta_{e}$ of less than 90°, which is characteristic of the lyophilic interface (specifically referred to as hydrophilic when the liquid is water).

However, Young’s equation assumes that the solid surface is rigid, perfectly smooth, and chemically homogeneous. In reality, most surfaces exhibit microscopic roughness and compositional heterogeneity, making it challenging for this idealized model to accurately describe real-world wetting behavior. To address this limitation, Wenzel modified Young’s equation by introducing a roughness parameter (R), defined as the ratio of the actual surface area to its projected area (R>1), to account for the impact of surface roughness on the contact angle (Figure 2b) [23,24]:

(2) $$\cos\theta_{r}=R\cdot \cos\theta_{e}$$

where $\theta_{r}$ represents the apparent contact angle on a rough surface. According to the Wenzel model, surface roughness effectively amplifies wettability characteristics: For hydrophilic materials ($\theta_{e}<90^{\circ }$), increased surface roughness reduces the apparent contact angle and enhances surface hydrophilicity. Conversely, for hydrophobic materials ($\theta_{e}>90^{\circ }$), elevated roughness increases the apparent contact angle and results in amplified hydrophobicity.

Building on this, Cassie and Baxter extended the model by considering the heterogeneity of surface chemical composition and the presence of air pockets at the solid–liquid interface. They proposed a weighted-average approach to describe the contact angle on composite surfaces (Figure 2b) [25]:

(3) $$\cos\theta =\sum_{1}^{n}f_{i}\cdot \cos\theta_{fi}$$

where $\theta_{fi}$ represents the contact angle of each pure component and $f_{i}$ is the fractional surface coverage of each component. This model enables accurate predictions of surface composition ratios based on observed contact angles. When a droplet interacts with a rough surface containing both solid and air regions, the proportion of liquid–solid and liquid–air contact can be expressed as $f_{1}$ and $f_{2}$, respectively, where $f_{1}+f_{2}$ = 1.

From these contact angle theories, it is evident that macroscopic wetting behavior is governed by both structural factors (surface roughness) and molecular-scale interactions. Consequently, modern strategies for tailoring surface wettability focus on two main approaches: the regulation of micro/nanostructure morphology and molecular-level chemical modifications.

For example, the research team led by J. Lei utilized thermoresponsive poly(N-isopropylacrylamide) (PNIPAAm) to achieve reversible transitions between superhydrophilicity and superhydrophobicity through temperature control [26]. This transition follows a mechanism similar to the Cassie–Baxter wet state observed in porous surfaces [25,27,28]. At lower temperatures, the extended PNIPAAm molecular chains form dense hydrogen-bonding networks that promote water penetration into surface microstructures, resulting in superhydrophilicity. At higher temperatures, intrachain hydrogen bonding dominates, leading to molecular contraction and the trapping of air within the rough surface, thereby inducing a hydrophobic state.

However, these systems, which rely on physical deformation, face intrinsic limitations. Due to the metastable nature of the hydrogen-bonding network, prolonged exposure to residual water molecules can degrade performance, while environmental contaminants may cause irreversible failure. This highlights the constraints of purely topological surface modifications and underscores the urgent need for more stable molecular engineering strategies to achieve long-term wettability control.

As liquid droplets spread, the movement of the water/air/solid three-phase contact line makes wetting an inherently dynamic process. It has been observed that the contact angle changes as a droplet moves on a solid surface [29]. When a droplet moves at a constant speed on a surface, the initially symmetrical droplet shape changes, and contact angle differences can be observed at the front and rear ends of the contact angle. The larger value represents the advancing angle ($\theta_{A}$) and the smaller value represents the receding angle ($\theta_{R}$). A larger $\theta_{A}$ value and a smaller $\theta_{R}$ value indicate that the droplet struggles more to expel air and wet the surface (Figure 2c). During the measurement process of real surfaces, the apparent contact angle θ obtained typically lies between $\theta_{A}$ and $\theta_{R}$ ($\theta_{R}<\theta <\theta_{A}$). For ideal surfaces described by Young’s equation, there is only one contact angle value. Therefore, in practical situations, the difference between the advancing angle and the receding angle ($\theta_{A}-\theta_{R}$) is defined as the contact angle hysteresis of the solid surface [30]. This can describe the degree of deviation from the ideal state and is directly caused by the thermodynamic metastability at the boundary of the solid–liquid–gas three-phase contact line, which is related to surface roughness, molecular reconstruction, and the non-uniformity of the surface chemical composition [31,32]. It can be said that $\theta_{A}$ and $\theta_{R}$ can more accurately characterize the true dynamic wettability of a surface.

Blake proposed a molecular dynamics theory (MKT) that describes competitive adsorption and desorption of liquid and air molecules near the three-phase contact line. The main parameters used to quantify the dynamics of wetting include the three-phase contact line velocity (U, the speed at which the liquid moves on the solid surface) and the dynamic contact angle ($\theta_{D}$, the angle between the tangent to the moving liquid interface at the three-phase line and the solid surface). During the wetting process, the three-phase line moves. Under certain system conditions and environments, there is a relationship between U and $\theta_{D}$. Generally, it is believed that as the contact line speed increases, the advancing dynamic contact angle (the contact angle when U>0) increases and the receding dynamic contact angle (the contact angle when U<0) decreases. That is, the dynamic contact angle depends on the speed and direction of the contact line movement. However, due to the existence of contact angle hysteresis, a stable and lower velocity of motion may not be measurable in experiments [33]. This process links macroscopic variables U and $\theta_{D}$ with microscopic thermodynamic variables and is represented by the following Formula (4) [34]:

(4) $$U=2{ҡ}^{0}\lambda \sin h\frac{\left[\gamma_{w/a}\left(\cos\theta_{D}-\cos\theta \right)\right]}{2nk_{B}T}$$

In the equation, ${ҡ}^{0}$ represents the molecular substitution frequency, λ is the distance between liquid molecules and adsorption sites on the solid surface, θ is the static contact angle, n is the number of adsorption sites p er unit area, $k_{B}$ is the Boltzmann constant, and T is the absolute temperature. Among these, ${ҡ}^{0}$ can be expressed as the wetting activation energy ${∆G}_{w}^{*}$, as follows:

(5) $${ҡ}^{0}=\left(\frac{k_{B}T}{h}\right)exp\left(\frac{{-∆G}_{w}^{*}}{N_{A}k_{B}T}\right)$$

where h and $N_{A}$ represent Planck’s constant and Avogadro’s number, respectively. When the distribution of adsorption sites is uniform and molecules only move between adjacent sites ($n\sim 1/2\lambda^{2}$), the only two unknown parameters values of λ and ${ҡ}^{0}$ in Equation (5) can be obtained by fitting experimental data to the equation.

It is clear that the dynamic wetting process described by molecular dynamics theory further highlights the critical role of molecular-scale interactions in dynamic wetting behavior, emphasizing the need to design surface hydrophilicity at the molecular level.

In this context, studying smooth nanostructured ion polymer surfaces, where roughness effects are negligible, allows us to isolate and systematically analyze the role of surface chemistry in wettability. Compared to neutral surfaces, ionic groups introduce additional environmental sensitivity, leading to unexpected variability of wetting behaviors under different conditions and expanding the possibilities for hydrophilic surface design. For instance, Wang et al. demonstrated that when a self-assembled monolayer (SAM) transitions from a neutral to an ionic state, surface polarity significantly increases, leading to a 40° decrease in the contact angle in air. In an oil environment, the hydration of ionic groups enhances surface hydrophilicity by two orders of magnitude [35]. This “chemical switching” effect originates from the synergistic interplay between ionic polymer polar groups and surface charges.

In summary, investigating the molecular mechanisms underlying surface hydrophilicity is crucial, and ionic hydrophilic surfaces serve as an ideal model system for such studies. This section will introduce the preparation methods for nanometer-smooth hydrophilic charged polymer surfaces and examine the molecular-scale factors influencing their wettability. By doing so, we aim to provide a foundational understanding of how to achieve hydration efficiency and stability in an ionic surface system.

### 2.1. Construction Strategies for Nano-Smooth Hydrophilic Ionic Polymer Surfaces

With the rapid evolution of contemporary materials science, surface modification techniques have emerged as a pivotal approach for augmenting material performance and broadening their technological applications. These methodologies can be broadly categorized into chemical and physical strategies, encompassing plasma treatment, surface grafting, and layer-by-layer (LbL) assembly via physical adsorption.

Chemical modification strategies typically refine surface properties by precisely tailoring the chemical composition and structural attributes of a material through controlled reactions. Plasma treatment exploits high-energy reactive species to induce surface functionalization, whereas surface grafting facilitates the covalent attachment of hydrophilic functional moieties onto the polymeric substrate, thereby enhancing wettability and adhesion characteristics. Conversely, physical approaches modulate surface properties through intermolecular interactions rather than chemical transformations. A notable example is the LbL assembly technique, which enables the fabrication of functional thin films via the sequential deposition of oppositely charged polyelectrolytes, yielding precisely controlled surface architectures.

This section provides an in-depth exploration of prominent surface modification strategies, delineating both chemical and physical paradigms while critically evaluating their respective advantages and limitations in optimizing polymeric surface hydrophilicity.

#### 2.1.1. Chemical Methods

**A.**  **Plasma Treatment**

**Figure 3:**
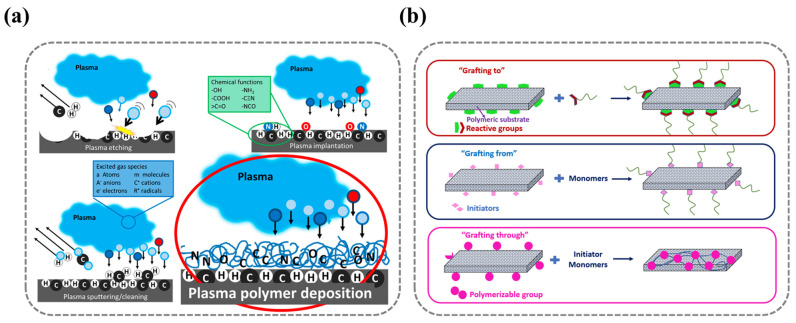
(**a**) Schematic of plasma treatment methods (including sputtering, etching, ion implantation, and polymer deposition processes). Ref. [51] Copyright from *MDPI*@2019. (**b**) Schematic of surface-grafting treatment methods (including grafting−to, grafting−from, and grafting−through approaches). Ref. [52] Copyright from *MDPI*@2021.

Plasma is a high-energy ionized gas phase composed of high-energy molecules, molecular fragments, ions, free radicals, and free electrons, which can be generated by methods such as magnetic fields, lasers, radiofrequency, electric fields, and microwaves. When plasma contacts a solid surface, it alters the material’s surface’s energy, resulting in changes to surface roughness and chemical properties. This can improve adhesion strength, surface and coating performance, and biocompatibility. Therefore, plasma has been widely researched and applied for surface modification [37]. The extent and functionality of plasma-induced surface modifications can be further refined through specialized techniques, including sputtering, etching, ion implantation, and plasma polymer deposition (Figure 3).

Specifically, when excited species in a plasma composed of inert gases (Ar, Ne, He, etc.) or reactive gases (O_2_, N_2_, NH_3_, CO_2_, etc.) collide with a solid surface, high-energy particles disrupt chemical bonds in polymers, causing surface-layer atoms to acquire enough energy to detach. This process, known as sputtering, is widely used for contaminant removal and serves as the fundamental principle of plasma cleaning. However, in cases where material loss extends deeper, leading to increased surface roughness, the process is termed etching. Additionally, reactive species within the plasma may directly engage with the substrate, initiating surface modifications. In this scenario, surface atoms generate radicals that interact with active plasma constituents, forming new functional groups that impart distinct chemical properties to the surface—an effect referred to as ion implantation.

For instance, Lai et al. conducted a comprehensive study on the surface properties of polycarbonate (PC), polypropylene (PP), and polyethylene terephthalate (PET) films subjected to microwave-induced argon plasma treatment. Their findings demonstrated that plasma exposure significantly enhanced hydrophilicity by altering surface roughness and functional group density. Experimental data further highlighted the critical role of C–O bond formation in improving polymer surface wettability [38]. Similarly, Junkar et al. [39] investigated the effects of O_2_ and N_2_ plasma treatments on PET polymers, revealing that O_2_ plasma treatment led to more pronounced improvements in both hydrophilicity and fucoidan adhesion. This enhancement was attributed to increased surface roughness, which provided additional adhesion sites for water molecules. Notably, plasma-induced functional groups are not necessarily all perfectly hydrophilic. Vesel et al. [40] pointed out that groups such as –COOH, –OH, –CO, and –NH_2_ promote hydrophilicity, whereas amides and lipids exhibit lower water solubility.

In a recent study, Zhu et al. developed an advanced dielectric barrier discharge (DBD) electrode array that effectively introduced hydroxyl groups onto PET surfaces, subsequently oxidizing them into carboxyl functionalities. This transformation markedly enhanced molecular polarity and induced complete carboxylation, endowing the surface with exceptional hydrophilicity—an approach well-suited for large-scale PET film processing [41]. Despite the remarkable efficiency of plasma treatment, surfaces modified via sputtering and ion implantation often exhibit near-surface gradients that deviate from thermodynamic equilibrium. Over time, hydrophobic recovery (passivation) may occur as polar groups undergo migration, reorientation, or diffusion, accompanied by charge leakage and deoxygenation, ultimately diminishing hydrophilicity [38,42]. Sharma et al. investigated the impact of helium (He) plasma treatment on the wettability of PC surfaces, finding that a 10 min exposure significantly lowered the contact angle, thereby enhancing hydrophilicity [43]. However, prolonged atmospheric exposure gradually diminished this effect, leading to a steady increase in contact angle.

Plasma polymer deposition presents a compelling solution to this challenge. Unlike other plasma-assisted surface modification techniques, this method induces the formation of chemically robust, stable organic coatings on the substrate. Hegemann et al. demonstrated that ultrathin (<10 nm) oxygen-functionalized plasma polymer films deposited on polycaprolactone (PCL) scaffolds exhibited permanent hydrophilicity [44]. In subsequent work, they optimized the plasma polymerization process by adjusting CO_2_ concentration and energy input, achieving a bilayer structure with a highly crosslinked base and a less crosslinked top layer. Aging studies revealed that, particularly for ultrathin gradient structures, the crosslinked sub-surface layer effectively hindered functional group reorientation, thereby mitigating hydrophobic recovery and oxidation effects [45].

In summary, plasma engineering offers a reliable, reproducible, and highly efficient approach to surface modification. Its applicability extends across diverse material classes, including metals, polymers, ceramics, and composites, and accommodates various sample geometries. Furthermore, real-time plasma diagnostics enable precise monitoring of plasma processes. Nevertheless, plasma treatment is not without limitations, including high equipment costs (due to reliance on vacuum systems sometimes), surface-limited modification effects (inapplicable to bulk or deep-layer structures), potential degradation of modified surfaces over time, the risk of material damage from high-energy particles, complex process parameter optimization, and safety concerns when handling hazardous gases.

**B.**  **Surface-Grafting Treatment**

Surface grafting is a sophisticated chemical strategy for anchoring ionic polymer chains onto solid substrates, thereby tailoring material properties with high precision. By introducing hydrophilic functional groups—such as hydroxyl (−OH), amino (−NH_2_), and carboxyl (−COOH)—onto a diverse range of substrates, including metals, glass, plastics, and silicon, surface grafting substantially enhances wettability and antifouling performance. Consequently, this approach has found extensive applications in biomedical implants, microfluidic systems, and antifouling coatings [46], offering an effective means to engineer surfaces with exceptional hydrophilicity, fouling resistance, and tailored interfacial properties.

As shown in Figure 3b, surface-grafting methodologies can be classified into three principal categories based on the mode of polymer attachment to the substrate: grafting to, grafting from, and grafting through.

The “grafting to” strategy entails the direct covalent bonding of pre-synthesized polymer chains—typically functionalized with reactive end groups such as hydroxyl or amino groups—to complementary functional groups on the substrate surface (e.g., silanol groups on silicon). For instance, Norma A. Alcantar and colleagues leveraged the condensation reaction between hydroxyl-terminated polyethylene glycol (PEG) and surface silanol groups to modify surface topology and chemistry, conferring enhanced hydrophilicity and biocompatibility [47]. However, this approach is inherently constrained by steric hindrance effects arising from the size of polymer chains, which impede the accessibility of reactive sites and limit the achievable grafting density. Additionally, poor control over polymer chain orientation often results in disordered surface structures, compromising functional performance.

In contrast, the “grafting from” technique involves immobilizing polymerization initiators onto the substrate, followed by in situ polymerization of monomers to generate high-density polymer brushes. Jiang et al., for example, functionalized a self-assembled monolayer (SAM) with an initiator on a gold surface, subsequently employing surface-initiated atom transfer radical polymerization (SI-ATRP) to graft β-sulfonate monomers, thereby fabricating zwitterionic coatings with superior antifouling capabilities [48]. This approach enables the formation of densely packed polymer brushes, offering greater control over surface functionality. However, the method is often hindered by intricate reaction pathways, which render the polymerization process challenging to regulate. Furthermore, precise control over molecular weight distribution remains a significant hurdle, alongside the tendency of monomers to undergo undesired self-polymerization.

The “grafting through” approach, on the other hand, exploits surface-initiated polymerization to construct crosslinked polymer networks, resulting in robust, high-density polymer brush layers [49]. Yamaguchi et al. employed SI-ATRP to synthesize and compare the wettability and antifouling properties of nonionic and ionic polymer brushes, demonstrating that crosslinking substantially enhances mechanical durability and environmental stability [50]. Despite its advantages, this method necessitates highly controlled polymerization conditions, including specific catalysts and stringent temperature regulation. Additionally, the resultant polymers often exhibit broad molecular weight distributions, necessitating further post-processing to achieve uniformity and reproducibility.

While surface-grafting techniques hold immense promise for advancing material surface engineering, their widespread implementation remains impeded by several practical limitations, including high production costs, extended reaction times, and inherent methodological constraints. Overcoming these challenges is paramount for unlocking their full potential in next-generation surface modification applications.

#### 2.1.2. Layer-by-Layer Physical Adsorption Method

Given the intrinsic instability of plasma-treated hydrophilic surfaces, alongside the prohibitive costs, complexity, and unpredictable reaction sites inherent to chemical grafting methods, there has been a concerted effort to identify scalable, industrially viable techniques for the fabrication of nanoscale smooth hydrophilic surfaces. Since the seminal work of Iler in 1966, which demonstrated the formation of uniformly thick silica and alumina films on glass substrates [53], the construction of polyelectrolyte multilayers (PEMs) via physical adsorption has emerged as a widely adopted, cost-effective, and operationally straightforward methodology.

To date, the primary physical adsorption techniques employed for the fabrication of hydrophilic polyelectrolyte coatings are based on Langmuir–Blodgett (LB) technology [54] and layer-by-layer (LBL) assembly techniques [55]. LB technology involves the transfer of amphiphilic molecules from an air–water interface to a solid substrate, resulting in the formation of monolayers or multilayers, followed by subsequent deposition or modification. In contrast, the LBL approach entails the sequential deposition of polyelectrolytes with opposing charges, utilizing non-covalent interactions to assemble nanoscale bilayers or multilayers, thereby affecting surface modification.

While LB technology is based on the transfer of amphiphilic molecules from the air–liquid interface to the solid substrate to form monolayers or multilayers, it is constrained by the requirement for stable monolayer formation at the air–liquid interface, thus limiting its flexibility in terms of design and practical applications. Conversely, the LBL method affords precise control over the film thickness at the nanoscale by tailoring assembly parameters such as polyelectrolyte type and deposition conditions. Moreover, LBL technology is compatible with a wide variety of substrates, including glass, ceramics, inorganic materials, and polymers, rendering it one of the most widely utilized coating techniques across diverse fields such as optics, energy, catalysis, separation, and biomedicine [56,57,58].

**Figure 4:**
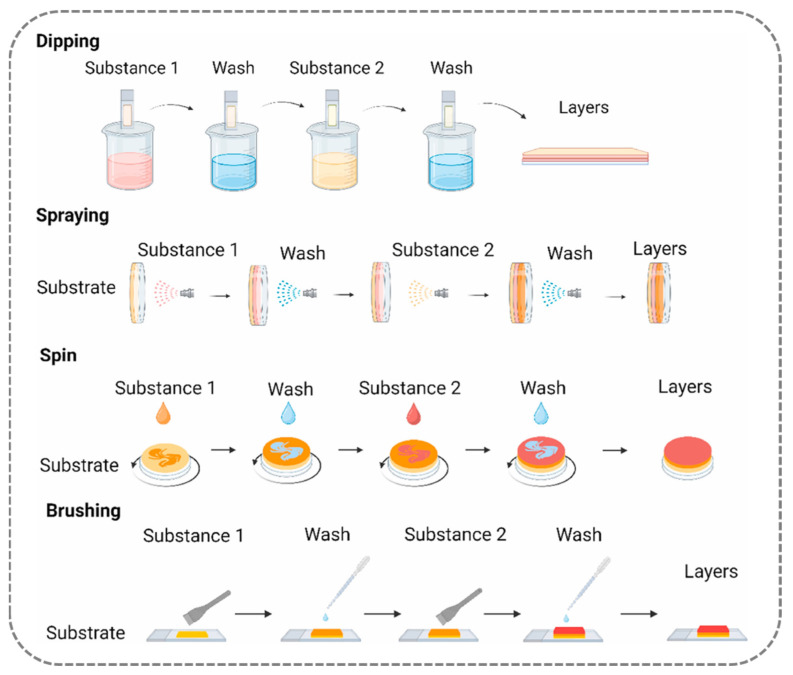
Schematic illustration of the common preparation methods for construction of layer-by-layer self-assembled films (including dipping method, spraying method, spin-coating method, and brushing method). Ref. [82] Copyright from *Elsevier*@2024.

**Figure 5:**
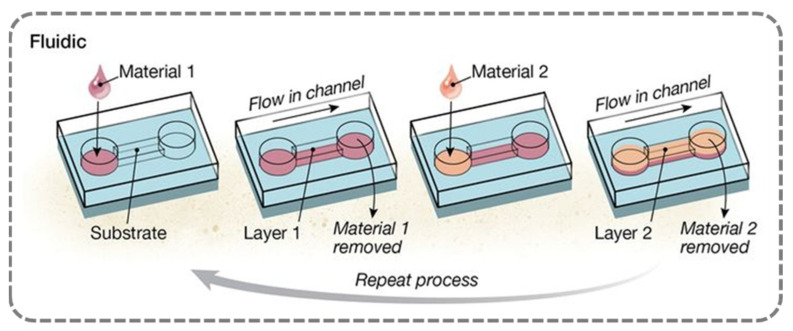
Schematic of preparation method for layer-by-layer self-assembled films (fluidic assembly). Ref. [55] Copyright from *AAAS*@2015.

This section will delve into several commonly used LBL techniques, as illustrated in Figure 4 and Figure 5. The LBL method, leveraging non-covalent interactions between polyelectrolytes, offers a relatively simple approach with enhanced design flexibility and versatility, solidifying its position as the prevailing methodology for the fabrication of nanoscale smooth hydrophilic surfaces.

**A.**  **Immersion Assembly Method: Electrostatic Self-Organization Through**

The immersion assembly method, central to layer-by-layer self-assembly technology, is predicated on the alternating adsorption of oppositely charged polyelectrolytes to construct nanoscale thin films. The typical process consists of three sequential stages: first, the substrate is immersed in a cationic polyelectrolyte solution, imparting a charge to its surface; excess polyelectrolyte molecules, which lack intermolecular interactions with the solid surface, are then removed through rinsing with deionized water. The substrate is subsequently transferred to an anionic polyelectrolyte solution for the deposition of the oppositely charged layer, culminating in the formation of a stable multilayer structure. By repeating this cycle, the film thickness can be precisely controlled at the nanometer scale by a simple process, rendering this approach applicable to a wide array of substrates, from planar materials to complex three-dimensional geometries [59].

Xu et al. demonstrated the engineering feasibility of this technique through biomimetic design: they sequentially deposited polydopamine (PDA) and polyethyleneimine (PEI) onto the surface of polypropylene microfiltration membranes, significantly reducing the modification time from the conventional 12 h required for a single PDA layer to just 3 h [60]. The optimized PDA/PEI composite coating reduced the water contact angle from 102° to 25° and increased water flux by nearly tenfold, showcasing remarkable oil–water separation performance. The non-covalent interaction mechanism underlying immersion assembly—namely, the synergistic effects of electrostatic interactions and hydrogen-bond networks—facilitates the deposition of uniform films on substrates of diverse shapes, including particles and irregularly shaped bulk materials, thus it has the advantage of offering universal applicability.

Nevertheless, processing large substrates in a single batch requires substantial solution volumes, and each deposition-rinsing cycle can take several minutes, causing traditional immersion assembly to encounter efficiency limitations when scaled up for industrial applications. In response, various automated strategies have been developed [61,62]. Kotov’s team introduced a continuous-flow deposition technique: by meticulously controlling the solution flow rate and contact time, they transformed diffusion-driven random adsorption into interface-dewetting-controlled directional assembly, enhancing the deposition rate per layer by approximately 30 times. The refined process not only preserves the precision of nanometer-scale thickness control but also induces the fractal growth of carbon nanotubes, resulting in a fishnet-like reinforcement structure that enhances the tensile strength of the film [63]. This high-throughput fabrication strategy represents a significant breakthrough for the industrialization of immersion assembly; however, meticulous fluid dynamics design remains necessary to regulate residual solution and prevent cross-contamination.

**B.**  **Spray Coating Assembly Method: Rapid Atomized Deposition and Uniformity Limitations**

The spray-coating technique, initially developed within the industrial coating domain, is predicated on the principle of rapidly constructing multilayer films by alternately spraying polyelectrolyte solutions of opposite charge onto a vertical substrate surface [64,65]. In contrast to conventional immersion methods, this technology offers an optimization scheme of substantial efficiency gains: through vacuum-assisted solvent evaporation, the deposition time for a single layer is reduced from several minutes to a mere 6 s, while only requiring a low-concentration solution of 0.1–0.5 mg/mL (corresponding to just 2–10% of the quantity used in immersion methods), significantly lowering production costs [64,66]. Groundbreaking research by Schlenoff et al. demonstrated that multilayer films fabricated by spray coating with the PDDA/PSS system exhibit morphology, uniformity, and chemical properties that are highly comparable to those produced via immersion techniques, thereby validating the feasibility of transitioning between these methods [67].

The fundamental advantage of this technique also lies in its ability to enable the formation of diverse structural configurations, and its precise control over surface morphology significantly broadens its potential applications [65,68]. For instance, the Criado team successfully integrated ferrite nanoparticles into a sodium alginate/chitosan matrix, producing enhanced films with a Young’s modulus of 2.8 GPa and a 300% improvement in adhesion strength, despite an increase in surface roughness from 20–80 nm to 50–200 nm due to nanoparticle incorporation [69]. This underscores that spray coating is not only applicable to pure polymer systems but also facilitates the precise co-deposition of inorganic/organic composite materials, with surface characteristics that can be directed and designed through careful modulation of components [70]. Furthermore, its ability to coat non-planar surfaces overcomes the geometric limitations inherent in traditional spin coating, thus enabling uniform deposition on large-area curved substrates [68].

Nonetheless, spray coating faces significant challenges in industrial applications. The influence of gravity results in droplet accumulation on the substrate surface, often leading to uneven local film thicknesses. Moreover, the random aggregation of excess droplets can induce irregular surface patterns, severely limiting the microscopic uniformity of the films. Although the performance advantages of this technique have been established at the laboratory scale [71,72], the process’ inherent instability restricts its widespread adoption in industrial contexts, with relatively few practical implementations to date.

**C.**  **Spin Coating Assembly Method: Centrifugal Force-Induced Uniform Deposition and Substrate Morphology Limitations**

Spin coating is a technique that leverages centrifugal force to achieve the rapid and uniform deposition of polyelectrolytes [73]. The method primarily operates in two modes: one involves directly dispensing the solution onto a rotating substrate (direct deposition method) [74], while the other first applies a static pre-coating, followed by high-speed rotation [75]. During the spinning process, the interaction between centrifugal and viscous forces drives the solution to spread across the surface, while electrostatic interactions orchestrate the orderly alignment of polymer chains, thereby facilitating the formation of thin films within seconds and improving the production efficiency greatly.

A distinguishing feature of spin coating is the inverse relationship between rotation speed and film thickness, making it a critical technique for fabricating multilayer structures [74]. This effect is particularly prominent for small molecules, where their high mobility influences changes in both porosity and roughness [76,77]. Stana et al. underscored the precision of this method in the preparation of TMC/alginate multilayer films, where surface charge and hydrophilicity were meticulously controlled to fine-tune the encapsulation and release dynamics of ketoconazole [78].

Presently, automated injection systems along with programmable spin-coating machines have rendered spin coating a viable option for the industrial-scale production of polyelectrolyte multilayer films; for example, it is commonly used in semiconductor photolithography. However, its dependence on ion concentration to control coating characteristics restricts its applicability to flat substrates, as it exhibits diminished performance on surfaces with more complex topographies [55]. Additionally, a mismatch between solution viscosity and rotational speed can introduce defects—high-viscosity solutions tend to accumulate at the edges, while low-viscosity conditions may lead to film rupture. Furthermore, an increase in ionic strength or inappropriate rotational speeds can disrupt the uniform distribution of the solution, resulting in thickness gradients, thus necessitating precise optimization of the process parameters.

**D.**  **Brush-Coating-Assisted Assembly Method: Direct Solution Deposition and Enhanced Molecular Interactions**

The brush-coating-assisted assembly method is a technique in which charged molecules from a solution are directly brushed or coated onto charge-sensitive surfaces, thereby facilitating the formation of layer-by-layer self-assembled films [79]. This process primarily depends on the intermolecular forces between the solution molecules and the surface, followed by the interactions generated between polyelectrolyte polymers, enabling their attachment to the surface and the progressive formation of a thickness-controlled layered structure.

A significant advantage of the brush-coating technique lies in its ability to accelerate film production while minimizing material waste. For instance, Iqbal et al. demonstrated the use of this method to produce tannic acid and collagen nanofilms, which were employed to promote the development of human muscle fibers [80]. The method is both versatile and straightforward, capable of forming fiber-aligned surface morphology under acidic pH conditions. Furthermore, compared to traditional immersion-based LBL assembly, brush coating facilitates the rapid formation of effective separation layers. Li et al. introduced an innovative electro-brush-assisted interlayer stacking (LBL) assembly technique, which was used to prepare lignin/polyelectrolyte membranes exhibiting outstanding separation performance, with an anion rejection rate exceeding 90% and a permeability of 66.3 L·m^−2^·h^−1^ [81]. While this approach reduced material waste by 80% compared to immersion methods, the practical application of brush-coating may be compromised by variability in uniformity due to the operator’s skill, environmental conditions, and tool precision, leading to surface inconsistencies that could negatively impact the final material performance. In addition, weak intermolecular forces result in weak binding layers. As a result, reports regarding this method have become less frequent.

In summary, while the brush-coating-assisted assembly method offers notable advantages in terms of accelerating film production and reducing material waste, its practical application remains constrained by the uniformity of operation and environmental conditions. Further optimization is necessary to enhance its stability and reliability.

**E.**  **Fluidic Assembly Method: Microchannel-Based Dynamic Adsorption and Real-Time Monitoring**

The fluidic assembly method is a technique used to prepare multilayer films by alternately adsorbing oppositely charged fluids within microchannels. This process can be executed by directly coating the channel walls or by depositing onto substrates fixed within the channel [83]. The technique typically involves the sequential driving of polymer solutions and wash fluids through pipelines, capillaries, or microfluidic networks [84,85]. Fluid transport can be achieved through pump pressure, capillary force, or rotation, or alternatively, by pipetting or static incubation. It is important to note that when the polymer solution remains in static contact with the substrate for extended periods (e.g., more than 10 min) [86], the impact of fluid flow on the growth of polyelectrolyte multilayer films diminishes, rendering the process similar to immersion assembly. Under typical conditions, when using pump- or vacuum-driven fluids, a film of approximately 1.5 nm thickness can be deposited within 5 to 10 min [87].

Capillary-driven fluidic assembly involves introducing the solution at the entrance of the channel, where capillary forces spontaneously drive the polymer solution through the microchannel, followed by the rotation of the substrate to remove excess liquid. This method can form a uniform film layer with a thickness of 1.2 nm in just 2 min [88]. The capillary-driven approach offers the advantage of simplicity, as it does not require external equipment, but its applicability is limited to small-volume channels and presents challenges in dynamic flow control.

In the context of fluidic assembly, the dissipative quartz crystal microbalance (QCMD) is an indispensable complementary tool. It enables real-time monitoring of the acoustic properties and growth dynamics of polyelectrolyte multilayer films [89]. Research has shown that the higher the concentration of the polymer solution, the thicker the resulting film. Contact time (rather than flow rate) is the key parameter that governs polymer adsorption efficiency under flowing conditions. Plunkett’s team innovatively utilized QCMD to measure resonance frequency shifts in non-deuterated and deuterated solvents, thereby enabling precise determination of the molecular weight and the number of bound water molecules in the film [90]. This method, which aligns seamlessly with the XPS mass analysis of dried films, provides reliable data on the water absorption capacity of polyelectrolyte films.

To sum up, the unique value of the fluidic assembly method lies in its spatial adaptability and cost-effectiveness. It enables uniform coating on surfaces, such as the interior of capillaries, which are difficult to access using traditional methods, and provides a novel strategy for localized patterning of coatings. Additionally, the method significantly reduces reagent consumption and lowers production costs in large-scale industrial applications. By employing both physical and chemical approaches, we can modify the chemical properties of solid surfaces to control their wettability. Fluidic assembly stands out due to its lack of reliance on complex equipment and its ability to control surface modification by adjusting the type of polyelectrolytes and preparation conditions. As such, this method remains an ideal choice for the investigation of polymer films with customized wettability and release properties. However, its limitations include the need for specialized equipment to construct microfluidic systems and relatively complex operations, which necessitate the operator’s specialized knowledge and expertise.

In summary, the analysis of various surface modification techniques demonstrates that the preparation of ionic polymer surfaces is relatively straightforward, with multiple available methods providing an efficient approach for surface modification across a wide range of applications. Furthermore, surface properties can be precisely controlled by selecting appropriate preparation methods and raw materials, making the use of ionic polymer surfaces for achieving hydrophilicity entirely feasible. The different preparation methods and their specific processes are summarized in Table 1.

Notably, the layer-by-layer (LBL) assembly physical adsorption method stands out as a simpler and more cost-effective alternative to chemical modification techniques. This approach relies on the straightforward physical assembly of charged polymer surfaces, eliminating the need for complex chemical reactions and expensive processing. Additionally, by optimizing process parameters, raw material consumption can be minimized, further reducing production costs and making LBL a highly practical solution for achieving hydrophilic surfaces.

Beyond its economic and operational advantages, LBL technology also offers superior controllability, making it particularly suitable for modifying single-charge molecular surfaces and enabling more precise investigations of surface properties. For instance, Whitesides et al. utilized surface grafting to fabricate four types of alkanethiol self-assembled monolayers (SAMs) on gold—positively charged, negatively charged, 1:1 positive-negative, and zwitterionic surfaces—to evaluate fouling resistance based on different ionic group terminations [91]. In comparison, Wang et al. employed the LBL assembly method using PDDA and PSS, with XPS data confirming that the resulting multilayer film contained only one type of counterion. By simply adjusting the number of adsorption layers, they were able to generate different surface properties and study their self-cleaning behavior [19].

**Table 1:** Summary of construction strategies for nano-smooth hydrophilic ionic polymer surfaces.

Type	Method	Technology	Principle	Scope of Application		Advantage	Disadvantage	Example
Chemical Method	Plasma	Sputtering	React with high-energy reactive substances such as ions, electrons, and free radicals	Metals, polymers, ceramics and composites.	Modify and clean surfaces	Simple and fast High efficiency Shape of substrate is Unlimited	Modified surfaces are unstable sometimes. Require specialized equipment/process control Limited to the surface of the material Damaging material potentially	[41,43,92,93,94,95,96]
		Etching			Increase surface roughness and patterning			
		Implantation			Implanting new functional groups			
		Deposition			Formation of thin organic coatings			
	Grafting	Grafting onto	Chemically bonding active-end polymer chains to substrates with active groups	Metal, glass, plastic and silicon		Suitable for pre-synthesized polymers	Low graft density Poor structural order	[46,47,52,97]
		Grafting from	Polymer growth initiated by substrate-bound initiator			Enables high-density polymer brushes.	Complex process Risk of monomer self-polymerization	[48,52,97]
		Grafting Through	Surface-initiated polymerization				Harsh reaction conditions Require post-processing	[50,52,97]
	Layer-by-Layer Assembly	Immersion	Alternating deposition via non-covalent interactions (electrostatic/van der Waals)	Most materials (metals, glass, plastics, nanoparticles)	Any shape of substrate	Simple process Compatible with automated procedures	Time-consuming High material consumption Cross-contamination risks	[59,60,61,62,63]
		Spray	Sequential spraying of oppositely charged polyelectrolytes		Large base substrates	Distinct layered film structure	Non-uniform film thickness Redundant pattern formation	[64,65,66,67,68,69,70,71,72,98]
Physical Method		Spin	Centrifugal and viscous forces spread the solution across the surface Electrostatic forces arrange polymer chains		Small flat substrates	Fast production speed Compatible with commercial Multilayer structures	Challenging for non-flat substrates non-uniform film thickness	[55,73,74,75,76,77,78,99]
		Brush	Non-covalent interaction between solution and surface or solution		Medium substrates	Precise thickness control Less raw materials required	Poor uniformity/repeatability	[79,80,81]
		Fluidic	Flow-driven alternating adsorption in microchannels		Complex geometries (e.g., capillary interiors)	Spatial adaptability Cost-effective	Limited flow control precision Requires microfluidic expertise Professional equipment	[83,84,85,86,87,88,89,90]

### 2.2. Molecular-Level Design of Surface Hydrophilicity (Influencing Factors): The Wettability of Solid Surfaces and Its Molecular Correlations

The wettability of solid surfaces is intrinsically linked to their functional performance in a myriad of applications. At the molecular level, wettability is predominantly governed by the molecular architecture of the surface’s ionic functional groups. Consequently, the ionic polar functional groups present on the solid surface play a pivotal role in modulating wettability and surface adhesion.

In addition to the polar groups on the outermost layer of the solid surface that engage directly with water molecules, the counterions and non-polar regions surrounding these groups also exert a significant influence on the polymer’s overall surface characteristics. When designing surface molecular structures, crucial factors such as the type of ionic groups, the proportion of non-polar groups, and the nature of counterions are vital in determining surface properties, particularly wettability. The interactions between various ionic groups and water molecules can either enhance or attenuate the surface’s hydrophilicity, while the three-dimensional conformation of the ionic groups on the outermost surface is subject to change in response to the surrounding environment, meaning that the surface of ionic polymers frequently undergoes dynamic molecular rearrangements in response to external conditions. This phenomenon is critical to the material’s stability and long-term performance. Moreover, the type of counterions and the associated hydration effects also have profound implications for surface wettability.

In this context, this section will review the factors that influence hydrophilicity on ionic polymer surfaces, with a focus on the molecular mechanisms underlying hydrophilicity, including the type of ionic groups, the impact of surface reorganization, and the nature of counterions on surface properties. A comprehensive understanding of these factors and their interrelationships is indispensable for the design of polymer materials with optimized performance.

#### 2.2.1. Types of Ionic Polar Functional Groups

The wettability of polyelectrolyte surfaces is largely governed by the interactions between water molecules and polymer chain segments, which, in turn, are dictated by the nature of the ionic polar functional groups present on the surface. Cheng et al. employed molecular dynamics simulations to investigate the underwater oleophobicity of self-assembled monolayers (SAMs) with different terminal functional groups. Their simulations demonstrated that the underwater oleophobicity of SAMs follows the sequence of methyl < hydrazine < oligoethylene glycol (OEG) < ethanolamine (ETA) < hydroxyl < mixed zwitterionic (NC_3_^+^/SO_3_^−^) groups. Furthermore, they found that alkyl trimethyl ammonium ions exhibit a higher water contact angle in air compared to surfaces containing sulfonic acid groups [100]. This finding underscores the strong correlation between surface hydrophilicity and the type of polar functional groups, indicating that the physicochemical properties of ionic groups on solid surfaces critically determine the strength and nature of interfacial interactions, thereby influencing wettability and hydrophilicity.

**Figure 6:**
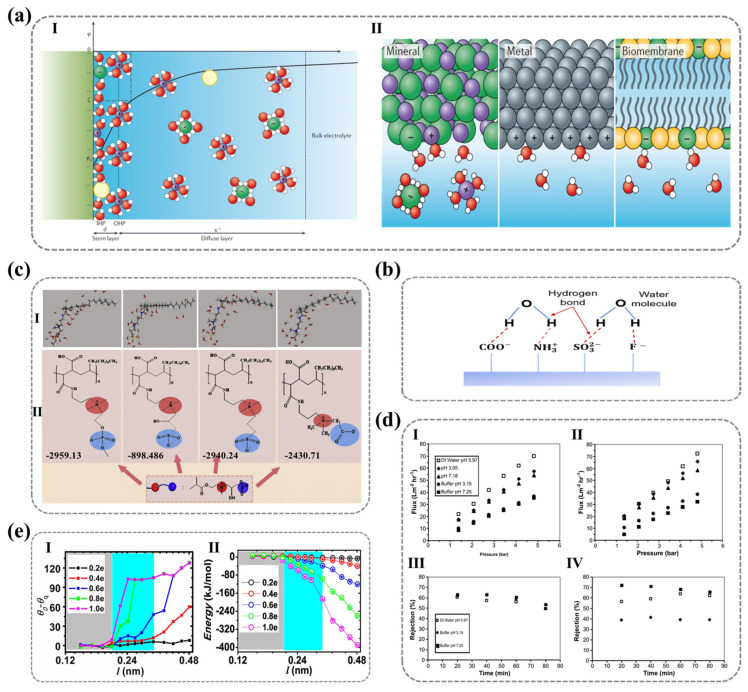
(**a**) Illustration of the electrostatic charge distribution at the solid−liquid interface based on the Gouy−Chapman–Stern model, highlighting the role of interfacial charge structures in governing hydration behavior. I. Schematic description of the Gouy-Chapman-Stern model (representing the inner Helmholtz plane (IHP), outer Helmholtz plane (OHP), the thickness of the Stern layer, and the thickness of the diffuse layer (Debye length)). II. Schematic of water interacting with charged surfaces via electrostatic forces (metal, oxide, biomembranes). Ref. [109] Copyright from *Springer Nature*@2021. (**b**) Schematic representation of hydrogen bonding interactions between water molecules and highly electronegative surface atoms (O, N, and F), emphasizing their contribution to enhanced hydrophilicity and hydration layer formation. (**c**) Correlation between molecular structure and hydration performance in zwitterion−grafted PVDF composite membranes, demonstrated through computational modeling (I) and experimental validation (II) of hydration energy, providing insights into the design of antifouling membranes. Ref. [105] Copyright from *Elsevier*@2020. (**d**) Comparative analysis of the pH−dependent filtration performance of unmodified and UV−grafted poly(acrylic acid) (PAA) membranes, focusing on filtrate flux (I, II) and glucose retention rate (III, IV) to assess the effectiveness of surface modification. Ref. [107] Copyright from *Elsevier*@2011. (**e**) Relationship between charge−induced wettability variations ((I) contact angle differences) and electrostatic interaction energy (II) as a function of dipole length, offering a deeper understanding of charge-driven water−solid interactions. Ref. [108] Copyright from *Springer Nature*@2012.

All ionic groups enhance hydrophilicity through electrostatic interactions between water molecules and the ionic polymer surface (charge attraction). Negatively charged polar groups on solid surfaces attract the hydrogen atoms of water molecules (which are partially positively charged), whereas positively charged groups attract the oxygen atoms of water molecules (which are partially negatively charged), as depicted in Figure 6a. The electrostatic interactions between surface charges and adsorbed polar water molecules modulate the interfacial water structure, a mechanism that can be fully explained by the electrical double-layer theory and DLVO theory. When an ionic surface acquires a net charge through ionization or ion adsorption, a spontaneous double-layer structure is formed at the interface, where counterions are tightly adsorbed in the Stern layer, while diffuse-layer ions follow a Boltzmann distribution, collectively establishing a potential gradient field. As illustrated in Figure 6a according to the Gouy–Chapman model, the decay of surface potential with distance can be mathematically expressed using Equation (6) [101,102]:

(6) $$\psi \left(x\right)=\psi_{0}\cdot exp\left(-\kappa x\right)$$

where $\psi_{0}$ represents the surface charge density and κ is a parameter related to the ionic strength of the solution, defined by the Debye–Hückel parameter in Equation (7):

(7) $$\kappa^{-1}=\sqrt{\frac{\epsilon k_{B}T}{2N_{A}e^{2}I}}$$

where ϵ denotes the dielectric constant of the solution, $k_{B}$ is the Boltzmann constant, T is the temperature, $N_{A}$ is Avogadro’s number, ee is the elementary charge, and I represents the ionic strength of the solution. This equation reveals the pivotal role of ionic strength (I) in regulating interfacial hydration, specifically by altering the thickness of the electrical double layer ($\kappa^{-1}\propto I^{-1/2}$). At low ionic strength, the potential decays more gradually, resulting in a thicker double layer and enhanced hydrophilicity; conversely, at high ionic strength, the Debye length decreases, leading to more rapid potential decay, compression of the double layer, and reduced hydrophilicity.

Moreover, within the potential gradient field, polar water molecules align their dipole moments with surface charges via electrostatic interactions, forming a hydration layer predominantly governed by hydrogen bonding networks. This structured interfacial water layer thermodynamically stabilizes the solid–liquid interface by reducing interfacial free energy, thereby enhancing wettability and significantly lowering the contact angle. The DLVO theory (Derjaguin–Landau–Verwey–Overbeek theory) further elucidates this phenomenon from the perspective of colloidal stability [103]. The DLVO theory describes the interplay of two primary forces that govern surface charge interactions—electrostatic repulsion and van der Waals attraction—offering a comprehensive framework for understanding the hydrophilicity of ionic surfaces. The total system potential energy ($V_{total}\left(d\right)$) is determined by the sum of electrostatic repulsion potential $V_{el}\left(d\right)$ and van der Waals attraction potential $V_{vd}\left(d\right)$) as described by Equations (8)–(10):

(8) $$V_{total}\left(d\right)=V_{el}\left(d\right)+V_{vd}\left(d\right)$$

(9) $$V_{el}\left(d\right)=\frac{64\pi \epsilon \rho_{r}{\gamma_{e}}^{2}k_{B}T\tan h^{2}\left(\frac{e\psi_{0}}{4k_{B}T}\right)exp\left(-\kappa d\right)}{\kappa^{2}}$$

(10) $$V_{vd}\left(d\right)=\frac{A}{12\pi D^{2}}$$

where d is the distance between two particles, $\rho_{r}$ is a parameter related to the ion concentration in the solution, $\gamma_{e}$ is the surface charge density, and A is the Hamaker constant representing the strength of the intermolecular attraction between surfaces.

Beyond electrostatic interactions, ionic groups can also attract water molecules through hydrogen bonding interactions. As shown in Figure 6b, atoms such as oxygen (O), nitrogen (N), and fluorine (F) are capable of forming hydrogen-bond networks with water molecules, thereby stabilizing the hydration layer. Consequently, polymer surfaces composed of functional groups with high charge density and greater electronegativity—those that readily form hydrogen bonds—tend to exhibit a more tightly bound hydration layer, thereby enhancing wettability. This phenomenon explains why cationic groups (e.g., -NH_3_^+^, -NR_3_^+^, and imidazolium groups) generally exhibit poorer wettability compared to anionic groups (e.g., -PO_4_^3−^, -SO_3_^−^, and -COO^−^). Since cationic groups have a weaker ability to form hydrogen bond interactions with water molecules, they primarily rely on electrostatic attraction rather than hydrogen bonding to orient water molecules. In contrast, zwitterionic functional groups, which contain both positive and negative charges, interact with different polarity regions of water molecules, establishing multiple binding sites. This leads to the formation of a more stable and compact hydration layer, yielding exceptionally high hydrophilicity. Moreover, unlike monovalent charged groups, zwitterionic functional groups can sustain hydrogen-bond networks within a span of several angstroms without significant disruption by surrounding water molecules. This unique property grants zwitterionic surfaces significant advantages in antifouling applications [104].

M. Maghami et al. [105] employed the fragment molecular orbital (FMO) method combined with pair interaction energy decomposition analysis (PIEDA) to study hydration and non-covalent interactions between water molecules and three distinct anionic functional groups. Their findings revealed that the hydration interactions between water molecules and sulfonate-based zwitterionic head groups are stronger than those with carboxylate and phosphate groups, as illustrated in Figure 6c. This behavior was attributed to the higher charge density of sulfonate groups and the steric hindrance of methyl groups in phosphoesters. Additionally, their research demonstrated that, due to the high local charge density of water molecules, their interactions with anionic groups are generally stronger than those with cationic groups [105].

Similarly, the aforementioned mechanisms also explain the environmental responsiveness of hydrophilicity in certain ionic functional groups. Changes in external factors can alter the charge state of polymeric groups, subsequently affecting polymer chain conformation and leading to variations in surface hydrophilicity. For example, the hydrophilicity of weak polyelectrolytes such as polyacrylic acid (PAA) is pH-dependent. When the pH exceeds the pKa of PAA, the carboxyl groups deprotonate and interact with water molecules through both hydrogen bonding and electrostatic interactions, thereby increasing surface hydrophilicity. Additionally, electrostatic repulsion between the charged groups causes the polymer chains to adopt an extended conformation. Conversely, when the pH is lower than the pKa, the carboxyl groups become protonated, and their interactions with water molecules are limited to hydrogen bonding, reducing hydrophilicity and inducing polymer chain collapse [106]. Himstedt et al. employed ultraviolet-induced radical polymerization to graft a nanoscale poly(acrylic acid) (PAA) layer onto nanofiltration membranes, finding that pH significantly influenced the membrane flux, retention rate, and selectivity for monosaccharides, disaccharides, and their mixtures, as shown in Figure 6d. This enhancement in membrane performance was attributed to the conformational changes in the grafted surface layer, driven by the protonation and deprotonation of acidic functional groups [107].

Moreover, despite the role of ionic polar groups in forming tightly bound hydration layers, spatial repulsion among water molecules may hinder hydrogen and oxygen atoms from approaching charged sites. This, in turn, weakens interactions between water molecules and surface ionic groups. Fang Haiping et al. demonstrated through molecular dynamics simulations that the dipole length of solid surface charge pairs plays a critical role in wettability. They identified a threshold dipole length below which its effect on wettability is negligible, as water molecules fail to “perceive” the dipole, rendering the surface hydrophobic despite the presence of polar groups. However, when the dipole length exceeds this threshold, increasing polarity progressively enhances surface hydrophilicity, as depicted in Figure 6e [108]. Thus, the dipole length of surface polar groups represents another key factor influencing surface hydrophilicity, further elucidating why different types of polar functional groups exhibit distinct hydrophilic properties.

#### 2.2.2. The Molecular Arrangement Pattern on Surfaces (Surface Reconstruction)

The inherent energy imbalance at solid surface atoms encourages the spontaneous rearrangement of molecules, a phenomenon particularly prominent when exposed to low-surface-energy environments (such as air). High-energy groups tend to migrate into the bulk phase to minimize interfacial energy. As early as 1987, Lee et al. employed transient contact angle measurements to study the surface dynamic modification of hydrophilic polymer systems, revealing a phenomenon of environmental-dependent polarity modulation. These surfaces exhibit enhanced polarity in high-polarity media (e.g., water) and a reduction in polarity when exposed to non-polar environments (e.g., air or oil), a behavior attributed to environment-induced molecular reorientation [110].

Although the hydrophilicity of ionized polymer surfaces primarily arises from the interaction between charged polar groups and water molecules, actual systems inevitably include non-polar groups (such as methyl or phenyl groups). When exposed to air, non-polar groups dominate by occupying energetically favorable positions, while polar groups reorient inward, minimizing exposure to reduce the overall surface energy, leading to an irreversible loss of hydrophilicity. This thermodynamic minimization of energy through molecular rearrangement establishes thermodynamic stability, resulting in the irrecoverable degradation of surface hydrophilicity even after prolonged water immersion. Thus, the environmental-induced changes in the three-dimensional conformation of polar groups lead to permanent hydrophilicity degradation, with molecular orientation becoming the dominant factor.

**Figure 7:**
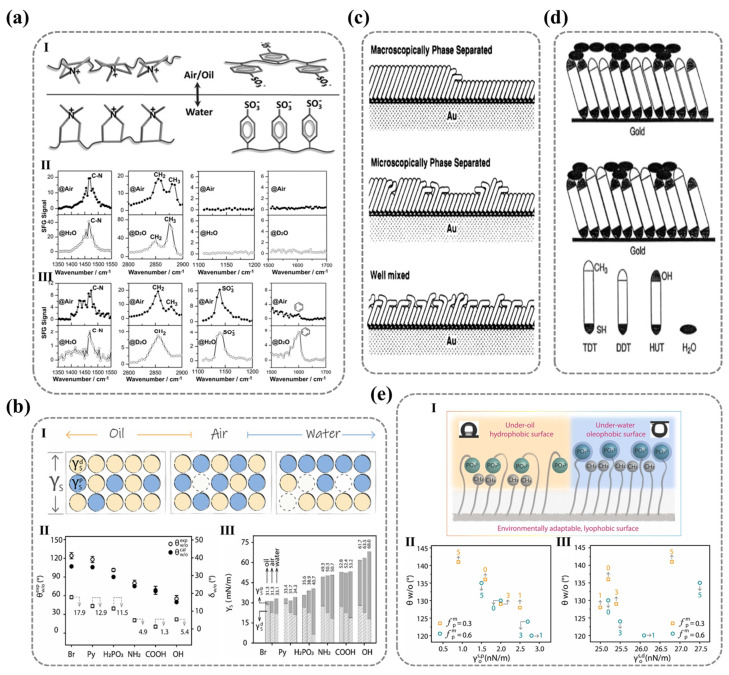
(**a**) Molecular rearrangement of the surface polar groups in (PDDA/PSS)_n_ multilayers driven by ambient polarity, revealed by SFG spectroscopy, highlights alkyl/phenyl signal inversion between air and water due to hydration effects. Ref. [19] Copyright from *Wiley*@2015. (**b**) Polar/dispersive surface energy ($\gamma^{p}$/$\gamma^{d}$) modulation of self-assembled monolayers (SAMs) across environments correlates terminal group chemistry with oil−water contact angle anomalies. Ref. [111] Copyright from *Elsevier*@2023. (**c**) Disordered molecular packing in mixed SAMs on gold, arising from chain−length mismatch, generates wettability-tunable heterogeneous surfaces. Ref. [116] Copyright from *ACS*@1992. (**d**) Chain−length−dependent hydration disruption in mixed monolayers demonstrates suppression of water layering by longer hydrophobic chains (C_14_SH). Ref. [117] Copyright from *ACS*@1991. (**e**) Polar/nonpolar group ratio ($f_{p}^{m}$) in binary SAMs governs oil−water contact angles and surface energy, identifying optimal compositions for antifouling applications. Ref. [35] Copyright from *Elsevier*@2024.

For single-component polymer surfaces, the three-dimensional restructuring behavior of polar groups depends on the stereochemistry of the polar groups themselves. Wang et al. employed layer-by-layer (LBL) assembly to fabricate polyelectrolyte multilayer films with terminal layers of polystyrene sulfonate (PSS) or poly(diallyl dimethylammonium chloride) (PDDA), revealing distinct environmental responsiveness between these two polar ionic functional groups. The PSS surface exhibited significant hydrophilic differences between air and water, while PDDA maintained stable wettability [19]. SFG vibrational spectroscopy characterization of surface molecular orientation reveals distinct configurations (Figure 7a): PDDA surfaces maintain cationic quaternary ammonium groups oriented toward the air phase in both air and aqueous environments, promoting surface hydration while exhibiting isotropic configurations. In contrast, PSS surfaces show strong hydrophobic benzene ring signals (weak sulfonate signals) in air, with this trend reversing in water—demonstrating their anisotropic nature that enables effective spatial separation. These observations conclusively determine environment-dependent molecular arrangements. Importantly, the results confirm that surface hydrophilicity is fundamentally governed by the integrated molecular architecture comprising both ionic groups and their adjacent nonpolar moieties.

Overall, surface wettability is not only determined by the intrinsic polarity of functional groups but is also critically influenced by their molecular structure—specifically, the spatial arrangement of adjacent non-polar groups and the dynamic reconfiguration of their environmental responsiveness. To further elucidate the relationship between surface wettability and molecular characteristics, Wang’s team [111] used the difference between the actual contact angle of water in the oil phase ($\theta_{w/o}^{exp}$) and the ideal contact angle ($\theta_{w/o}^{cal}$) as a parameter $\delta_{w/o}$ to measure the degree of reconstruction of the solid surface [112]. $\delta_{w/o}$ can be calculated using the following Formulas (11) and (12):

(11) $$\cos\theta_{w/o}^{cal}=\frac{\gamma_{w/a}\cos\theta_{w/o}^{exp}-\gamma_{o/a}\cos\theta_{o/a}^{exp}}{\gamma_{w/a}}$$

(12) $$\delta_{w/o}=\theta_{w/o}^{exp}-\theta_{w/o}^{cal}$$

where $\gamma_{w/a}$, $\gamma_{o/a}$, and $\gamma_{w/o}$ represent the interfacial tensions of water/air, oil/air, and water/oil, respectively. $\delta_{w/o}$ reflects the degree of deviation between the surface and the ideal surface, which remains unaffected by the environment. Therefore, the smaller the $\delta_{w/o}$ value, the smaller the degree of reconstruction of the solid surface. $\delta_{w/o}=5^{{}^{\circ}}$ can serve as a discriminating criterion for determining whether surface molecules undergo reconstruction. As shown in Figure 7b, the surfaces of SAM-NH_2_, SAM-COOH, and SAM-OH exhibit greater environmental stability compared to the surfaces of SAM-Br, SAM-Py, and SAM-H_2_PO_3_.

Then, Wang’s team emphasizes that the likelihood of surface molecular reconstruction in different environments correlates positively with the variation in the polar ($\gamma_{s}^{p}$) and non-polar ($\gamma_{s}^{d}$) components of surface energy (Figure 7b). Surface energy originates from incomplete atomic layers and asymmetric intermolecular interactions at solid surfaces, generating additional free energy at the interface. It is a key thermodynamic parameter for assessing surface physicochemical properties [113]. The magnitude of surface energy directly influences wettability: higher surface energy facilitates the wetting of liquids. In 1964, Fowkes proposed that surface energy consists of independent contributions from dispersive forces and polar forces [114], dividing surface energy (γ) into two main components: the non-polar (dispersive) component ($\gamma^{d}$) and the polar component ($\gamma^{p}$) with Equation (13):

(13) $$\gamma =\gamma^{d}+\gamma^{p}$$

To improve practical applicability, Owens and Wendt [115] developed a method (Equation (11)) in 1969 based on Fowkes’ theory, which separates the non-polar and polar components via contact angle measurements:

(14) $$\gamma_{l}\left(1+\cos\theta \right)=2\sqrt{\gamma_{s}^{d}\cdot \gamma_{l}^{d}}+2\sqrt{\gamma_{s}^{p}\cdot \gamma_{l}^{p}}$$

where $\gamma_{l}$ represents the total surface energy of the liquid and $\gamma_{s}^{d}$ and $\gamma_{l}^{d}$ are the non-polar components, while $\gamma_{s}^{p}$ and $\gamma_{l}^{p}$ are the polar components. By utilizing contact angle test data, one can quantitatively separate the non-polar and polar components of a solid surface, thereby assessing the potential for surface reconstruction and revealing the molecular mechanisms underlying the hydrophilicity of ionic polymer surfaces.

Beyond molecular reconstruction induced by nonpolar moieties adjacent to polymeric polar groups, molecular-scale roughness also correlates with surface reconstruction, consequently modulating surface hydrophilicity—a phenomenon predominantly observed in multicomponent self-assembled monolayers (SAMs). Whitesides and colleagues [116] studied the wettability of SAMs composed of non-polar alkyl thiol molecules with different chain lengths (C_12_SH and C_18_SH; C_12_SH and C_22_SH), finding that the monolayer of long-chain thiol molecules exhibited disorder outside the terminal monolayer of short-chain thiol molecules, as shown in Figure 7c. Ulman et al. [117] constructed a surface with two polar and non-polar functional groups at both ends of the molecule (C_12_SH and C_11_OH-SH; C_14_SH and C_11_OH-SH), observing that adding methylene groups to the hydrophobic portion (C_14_SH and C_11_OH-SH) induced molecular-scale roughness, significantly disrupting the interactions between polar groups (Figure 7b). Recently, Wang’s team [35] built a dual-component self-assembled film surface by maintaining the main chain length of thiol molecules fixed (with fixed polar terminal groups -H_2_PO_3_) and altering the main chain length of thiol molecules with non-polar groups (–CH_3_). As shown in Figure 7e, they found that in an oil environment, when the difference in molecular size (∆h) was small (1–3), the $\gamma_{s}^{p}$ value increased, while the $\gamma_{s}^{d}$ value remained low, indicating reduced surface hydrophobicity due to the diminished distance between non-polar groups (–CH_3_) and the interface. Polar groups (–PO_3_^2−^) did not undergo significant reorientation. However, when ∆h was larger (3–5), the $\gamma_{s}^{p}$ value decreased and the $\gamma_{s}^{d}$ value increased (Figure 7e), indicating significant internal reorientation of the polar groups, leading to surface reconstruction.

#### 2.2.3. Counter-Ion Effects

**Figure 8:**
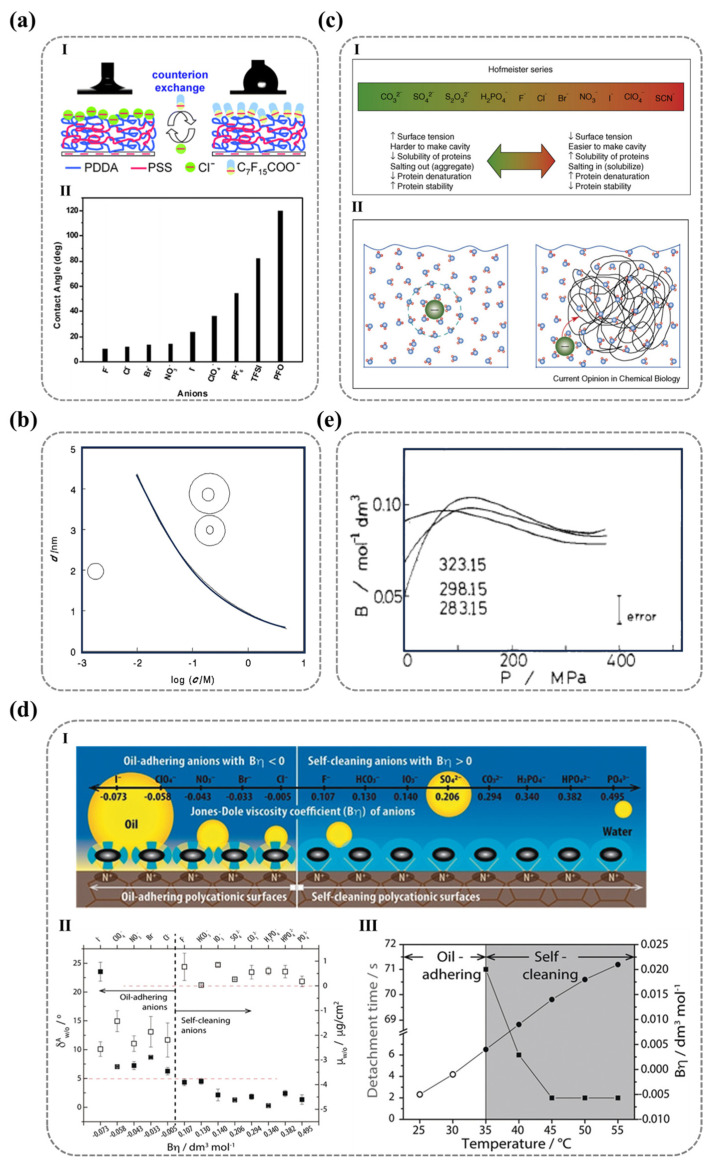
(**a**) Schematic of water contact angle changes in (PDDA/PSS)_n_ multilayer films in air, demonstrating the counterion−dependent wettability of polyelectrolyte multilayers. Ref. [118] Copyright from *RSC*@2009. (**b**) Relationship between the average interionic distance in solution and the logarithmic ion concentration (log *c*). The curve delineates the transition from isolated hydrated ions (**left**) to overlapping hydration shells (**right**). Ref. [119] Copyright from *ACS*@2009. (**c**) I. Hofmeister series ranking for different ions, illustrating ion−specific effects on macromolecular stability and hydration. II. Anion and water structure: Structured water beyond the first hydration shell of the anion is required to generate the structural formation and disruption effect (**left**); direct interaction between the anion and macromolecules in aqueous solution (**right**). Ref. [120] Copyright from *Elsevier*@2006. (**d**) Self−cleaning modulation of polyelectrolyte multilayers by tailoring counterions in PDDA−terminated films. The study links counterion selection to surface oil adhesion/removal behavior. Ref. [132] Copyright from *Wiley*@2020. (**e**) Pressure dependence of the ionic hydration parameter *B*_*η*_ for sodium chloride in aqueous solutions, revealing how external pressure alters ion–solvent interactions. Ref. [134] Copyright from *ACS*@1992.

For ionic functional groups, in addition to their intrinsic properties, the counter-ions also exert a significant influence on the wettability of solid surfaces. Wang et al. demonstrated this effect by modifying the counter-ions of the outermost PDADMAC layer in polyelectrolyte multilayers via ion exchange. As shown in Figure 8a, they observed that the contact angle varied from approximately 10° to 120°, providing direct evidence that the wettability of solid surfaces bearing ionic functional groups is closely correlated with the properties of their counter-ions [118]. When electrolytes dissolve in water, the resulting ions induce rearrangement of surrounding water molecules—a process termed hydration. The intense electric field generated by the ions organizes water molecules into a distinct solvation structure. This coordinated water assembly surrounding the ion constitutes the hydration shell, where the number of structured water molecules quantitatively defines the ion’s hydration capacity [119,120]. The hydration shell is generally categorized into primary and secondary hydration layers. The primary hydration layer is almost universally present around all ions, whereas the secondary hydration layer primarily surrounds highly charged ions. To better understand the structure of these hydration layers and their impact on water organization, it is essential to consider the spatial constraints within electrolyte solutions. In a solution with a concentration of c mol/L, the average distance d (nm) between ion centers can be expressed as follows [119]:

(15) $$d/nm=\left[10^{-3}\left(m^{3}/{dm}^{-3}\right)\times 1027\left({nm}^{3}/{dm}^{-3}\right)\right]/{\left[2c\left(mol{dm}^{-3}\right)N_{A}\left({mol}^{-1}\right)\right]}^{1/3}$$

In this equation, ion radius and solution concentration are the key parameters. The relationship between d and log⁡c for monovalent ions is depicted in Figure 8b, where the hydrated radii of Na^+^ and Cl^−^ are 0.378 nm and 0.458 nm, respectively [121]. At a concentration of 1 mol/L, the interionic distance d is 0.94 nm, allowing a water molecule to reside between the cationic and anionic hydration shells. At 2 mol/L, the average interionic distance is reduced to d=0.75 nm, leading to a tightly packed hydration shell with minimal free space for water molecules. This indicates that the hydration shells of NaCl remain intact even at high concentrations.

Due to its extensive hydrogen-bonding network, water is considered a highly structured liquid. When a surface bearing ionic functional groups is exposed to water, the counter-ions inevitably dissolve into the surrounding aqueous environment. The hydration of these counter-ions disrupts the native hydrogen-bond network of water, thereby introducing the concepts of “structure-making” and “structure-breaking” to describe the impact of ion hydration on hydrogen-bond networks. The former refers to ions with strong hydration abilities that enhance water structuring, while the latter pertains to weakly hydrated ions that disrupt the hydrogen-bonding network [122].

One of the most renowned frameworks for describing the influence of ions on water structure is the Hofmeister series, which provides a semi-quantitative ranking of ions based on their ability to alter water structuring [123,124]. For instance, ions with strong water-binding affinities can effectively compete with proteins for hydration, leading to protein precipitation due to dehydration. As illustrated in Figure 8c, the Hofmeister series ranks anions and cations according to their hydration effects as follows:

Hofmeister series for anions:

CO_3_^2−^ > SO_4_^2−^ > S_2_O_3_^2−^ > H_2_PO_4_^−^ > OH^−^ > F^−^ > HCO_3_^−^ > CH_3_CO_2_^−^ > Cl^−^ > Br^−^ > NO_3_^−^ > I^−^ > ClO_4_^−^ > SCN^−^

Hofmeister series for cations [125]:

(CH_3_)_4_N^+^ > (CH_3_)_2_NH_2_^+^ > K^+ ~^ Na^+^ > Cs^+^ > Li^+^ > NH_4_^+^ > Mg^2+^ > Ca^2+^

In this series, carbonate (CO_3_^2−^) exhibits the strongest structuring effect on water and the highest precipitation propensity, whereas thiocyanate (SCN^−^) is the most disruptive to water structure, sometimes even enhancing protein solubility. Grau et al. [126] used spectroscopic techniques to directly observe the water structure at the interface of heavy water (D_2_O), revealing that the molecular ordering of an octadecylamine monolayer in D_2_O follows the Hofmeister series, thereby validating its applicability to assessing ion hydration effects near polymeric surfaces.

Since hydrated ions alter the intrinsic structure of water, they inevitably influence the solution’s viscosity. The strength of ion–water interactions affects the viscosity of electrolyte solutions. Strongly hydrated ions tightly bind water molecules, significantly reducing fluidity and increasing viscosity compared to pure water. Gurney’s studies confirmed that some electrolyte solutions exhibit higher viscosities than pure water [122]. For example, at 25 °C, the viscosity coefficient *B*_*η*_ of the NaF solution is 0.205 M^−1^ [127], whereas certain salts, such as CsI, reduce water viscosity, with *B*_*η*_ reaching −0.258 M^−1^ at 0 °C [128]. The viscosity coefficient (*B*_*η*_) for electrolyte solutions was first introduced by Jones and Dole [109] through Equation (16) [129]:

(16) $$\frac{\eta }{\eta_{0}}=1+A_{\eta }c^{1/2}+B_{\eta }c$$

where η and $\eta_{0}$ represent the viscosities of the electrolyte solution and pure water, respectively. The term *A*_*η*_ reflects ion–ion interactions, while *B*_*η*_ quantifies ion–solvent interactions. It is important to note that this equation is most applicable to infinitely dilute ideal solutions with complete ion dissociation. Gurney further validated the impact of ions on water viscosity based on reagent-based measurements [130]. The *B*_*η*_ coefficient serves as an effective indicator of ion hydration strength:

Ions with *B*_*η*_ > 0 enhance water structuring, indicating strong hydration.

Ions with *B*_*η*_ < 0 disrupt water structuring, leading to a more loosely bound hydration shell and weaker hydration ability.

As mentioned in the introduction of Section 2, dynamic contact angles can more accurately reflect the wetting behavior between different phases. Compared to the receding angle, the advancing angle better reflects the state of surface molecules in the de-wetting phase [131]. Thus, Wang et al. correlated *B*_*η*_ values with the deviation ($\delta_{w/o}^{A}$) between theoretical and actual advancing angles in oil and water–oil displacement efficiency ($\mu_{w/o}$) to predict self-cleaning surface performance [132]. Here, $\mu_{w/o}$ quantitatively describes the degree of de-wetting of oil from the solid surface under the action of water, and it can be calculated using Equation (17):

(17) $$\mu_{w/o}=\left(m_{o/a}+m_{w/o}\right)-m_{w/a}$$

where $m_{o/a}$, $m_{w/o}$, and $m_{w/a}$ correspond to the mass of oil adsorbed in air, the mass of water adsorbed in the oil phase, and the mass of water adsorbed in air by the polyelectrolyte multilayer films with a PDDA-X surface, respectively. When water cannot completely replace the oil on the contaminated surface, $\mu_{w/o}<0$. Conversely, when water can completely replace the oil, $\mu_{w/o}>0$. From the data shown in Figure 8d, for the sticky oil PDDA-X surface, *B*_*η*_ $<0{,\;\delta }_{w/o}^{A}>5^{{}^{\circ}},\;\mu_{w/o}<0$, while for self-cleaning surfaces, *B*_*η*_ $>0,\;\delta_{w/o}^{A}<5^{{}^{\circ}},\;\mu_{w/o}>0$. Therefore, $\delta_{w/o}^{A}=5^{{}^{\circ}},\;\mu_{w/o}=0$ can also be considered the threshold to determine whether the surface hydration layer is complete.

Wang et al. also pointed out the crucial role of temperature in this process [132]. For PDDA-Cl surfaces with *B*_*η*_ being negative, as the environmental temperature increases above 35 °C, *B*_*η*_ gradually becomes positive, which enables the oil adhesion coating to exhibit self-cleaning properties in water (Figure 8d). This is because *B*_*η*_ changes with temperature. Generally, for ions that establish a water structure [133], *B*_*η*_ > 0 and *dB*_*η*_/*dT* < 0, while for ions that disrupt the water structure, *B*_*η*_ < 0 and *dBη*/*dT* > 0.

Furthermore, *B*_*η*_ is also influenced by pressure. As illustrated in Figure 6e, Sawamura’s research shows that at 10, 25, and 50 °C, the *B*_*η*_ value of NaCl varies with pressure. Below 100 MPa, *B*_*η*_ increases with pressure at 10 and 25 °C, while remaining relatively constant at 50 °C. At higher pressures, *B*_*η*_ decreases across all temperature ranges due to reduced structural water under extreme compression [134].

The counter-ion effect is thus crucial in modulating surface hydrophilicity and is also of paramount importance in biological surface interactions and molecular biology, offering deeper insights into fundamental biological processes.

## 3. Molecular Design-Guided Applications for Hydrophilic Surfaces

In Section 2.2, we explored how the type, configuration, and counterion interactions of ionic functional groups influence wettability when constructing hydrophilic interfaces. This provides theoretical guidance for molecular-level hydrophilic surface design. However, practical applications require tailoring the degree of hydrophilicity according to specific operational demands. For systems that require moderate hydrophilicity, materials can incorporate a higher proportion of hydrophobic components, where the primary design focus lies in optimizing the contribution of polar functional groups. Conversely, applications demanding ultra-hydrophilicity have a very low tolerance for hydrophobic components, necessitating a fully hydrated surface. This hydration threshold is typically achieved through a precise balance of hydrophilic and hydrophobic groups. When hydrophilic components reach sufficient hydration strength, they can effectively shield hydrophobic regions, thereby enabling super-hydrophilic behavior. Experiments by Ralston et al. demonstrated that even hydrophobic defects as small as 10–50 nm can impede the movement of the three-phase contact line [135]. This critical scale—far smaller than the liquid droplets used in conventional contact angle measurements—highlights the sensitivity of surface hydrophilic components. As discussed in the introduction of Section 2, the Cassie model can theoretically (Equation (3)) predict the wettability of chemically heterogeneous smooth surfaces: when $f_{i}$ refers to non-polar components and polar groups, respectively, macroscopic hydrophilicity emerges from the competition between polar group hydration and the suppression of hydrophobic defects.

**Figure 9:**
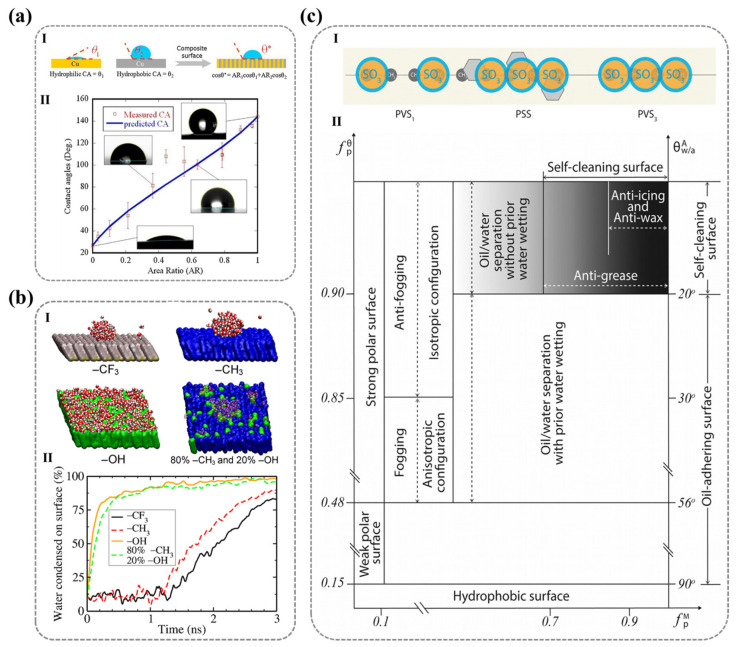
(**a**) Cassie–Baxter theory-guided design of composite copper surfaces achieves tunable hydrophobicity by tailoring hydrophilic/hydrophobic domain area ratios, validated through contact angle (*θ*) measurements and macroscopic wetting morphology analysis. Ref. [136] Copyright from *Elsevier*@2013. (**b**) Molecular dynamics simulations uncover surface chemistry-dependent water condensation dynamics, linking –CF_3_/–CH_3_/–OH group hydrophobicity to droplet contact angle stability and nucleation timelines under non-zero vapor baseline conditions. Ref. [137] Copyright from *AAAS*@2018. (**c**) Hydration-driven masking of nonpolar moieties (–CH_3_, –CH_2_–, and phenyl) adjacent to sulfonate groups governs surface hydrophilicity, while polar group packing density ($f_{p}^{\theta }$ and $f_{p}^{M}$) dictates environmental adaptability in PVS_n_-CS and PSS-CS architectures. Ref. [138] Copyright from *Wiley*@2023.

Both experimental and computational studies have validated the composition-dependent nature of hydrophilicity. Huang et al. engineered a tunable contact angle copper substrate by adjusting the ratio of superhydrophobic to superhydrophilic regions, achieving enhanced condensation heat transfer efficiency (Figure 9a) [136]. Wong et al. used molecular dynamics simulations to investigate mixed self-assembled monolayers (SAMs) with randomly distributed terminal −CH_3_ (80%) and −OH (20%) groups. The interpolated contact angles between pure methyl and hydroxyl surfaces revealed that water molecules selectively cluster around hydroxyl groups, providing direct evidence that hydrophilicity is dictated by composition (Figure 9b) [137].

Wang et al. systematically varied the adsorption cycle of sodium polyvinyl sulfonate (PVS) in (PDDA/PSS)_3.5_ + PVS_n_ multilayer films to control the density of free sulfonic acid groups ($N_{{SO}_{3}}^{F}$). They established two molecular descriptors of macroscopic hydrophilicity: the hydration density of polar groups ($f_{p}^{\theta }$) and the actual molecular packing density ($f_{p}^{M}$). The parameter $f_{p}^{\theta }$ derives from the Cassie model and relates the theoretical advancing contact angle $\theta_{w/a}^{A,cal}$ to molecular-scale hydration coverage according to Equation (18) [138]:

(18) $$f_{p}^{\theta }=\frac{\cos\theta_{w/a}-\cos\theta_{w/a}^{np}}{\cos\theta_{w/a}^{p}-\cos\theta_{w/a}^{np}}$$

where $\theta_{w/a}^{p}$ and $\theta_{w/a}^{np}$ represent the equilibrium contact angles of pure polar and nonpolar regions, respectively. Meanwhile, $f_{p}^{M}$ is obtained through geometric modeling of the spacing between polar groups (D) and $N_{{SO}_{3}}^{F}$ according to Equation (19):

(19) $$f_{p}^{M}=\frac{\frac{1}{2}\pi {R_{ion}}^{2}}{\frac{\sqrt{3}}{4}D^{2}}=0.05\cdot N_{{SO}_{3}}^{F}$$

where $R_{ion}$ denotes the projected area of the polar functional group (e.g., 0.14 nm for sulfonic acid). By integrating these parameters, the number of water molecules adsorbed per polar group ($N_{H2O}$) can be quantified:

(20) $$N_{H2O}=\frac{f_{p}^{\theta }-f_{p}^{M}}{f_{H2O}^{M,max}}\cdot n$$

where n represents the hydration shell requirement (e.g., 13.3 for sulfonic acid [139]), and $f_{H2O}^{M,max}$ is the maximum hydration contribution per polar group per unit area. When $f_{p}^{M}$ remains constant, a lower $N_{H2O}$ value indicates a stronger suppression of hydrophobicity by the polar groups.

As illustrated in the accompanying figures, increasing $f_{p}^{\theta }$ enhances antifouling and anti-fogging performance, while self-cleaning capabilities (e.g., oil–water separation, anti-icing, and anti-waxing) correlate closely with $f_{p}^{M}$. The hydrophilicity thresholds for specific applications are as follows: surfaces for oil–water separation and anti-fogging require $f_{p}^{\theta }>0.89$, $f_{p}^{M}>0.65$ whereas anti-icing and anti-waxing demand $f_{p}^{\theta }>0.93$, $f_{p}^{M}>0.85$. These thresholds reflect fundamental packing constraints: $f_{p}^{M}>0.65$ (the maximum density of two-dimensional random packing spheres [140]) ensures sustained hydration, while $f_{p}^{M}>0.90$ (the theoretical maximum density of two-dimensional close-packed spheres (hexagonal close packing)) achieves complete suppression of hydrophobicity. Thus, molecular design strategies must prioritize polar group–molecular configuration–counterion–water interactions to establish basic hydrophilicity while structuring the overall distribution of polar and nonpolar groups to achieve advanced functionalities.

This section systematically classifies hydrophilic surfaces based on application requirements and design complexity, providing concrete examples of molecular engineering. Through precise molecular-level design, hydrophilic surfaces hold vast potential in antifouling, anti-fogging, oil–water separation, anti-icing, and anti-waxing applications. Future research directions include further optimizing the ratio and distribution of polar and nonpolar groups, exploring novel ionic functional groups and their effects on wettability, and developing hydrophilic materials suited for extreme environments such as high temperatures and high pressures.

### 3.1. Tunable Hydration Layers for High-Flux Oil–Water Separation ($f_{p}^{M}>0.65$)

The growing importance of oil–water separation in industrial wastewater treatment has become increasingly evident, particularly in industries such as petrochemicals, mechanical manufacturing, and food processing, which generate substantial volumes of oil-contaminated wastewater. The improper treatment of oil-based pollutants can lead to severe water pollution, ecological degradation, and reduced water resource recovery efficiency. Consequently, there is an urgent need for the development of highly efficient, cost-effective, and environmentally sustainable separation technologies.

Traditional oil-removal methods, including combustion, skimmers, absorbents, and chemical dispersants, exhibit inherent limitations. Combustion generates harmful emissions, skimmers often retain excessive amounts of water, used absorbents require secondary treatment, and chemical dispersants pose marine toxicity concerns. In contrast, surface-functionalized membranes have emerged as a promising alternative due to their high permeability, enabling selective hydrophilic/oleophobic separation for rapid, continuous, and cost-effective oil–water separation.

The fundamental mechanism of hydrophilic surfaces in oil–water separation lies in their dual affinity for polar water molecules and their repulsion of non-polar oils. Upon contact with an oil–water mixture, water molecules rapidly adsorb onto the hydrophilic surface through hydrogen bonding or electrostatic interactions, forming a stable hydration layer. Conversely, due to the absence of polar interactions, oil molecules are repelled, facilitating efficient phase separation. This synergistic effect enables the recovery of high-purity water from emulsions.

**Figure 10:**
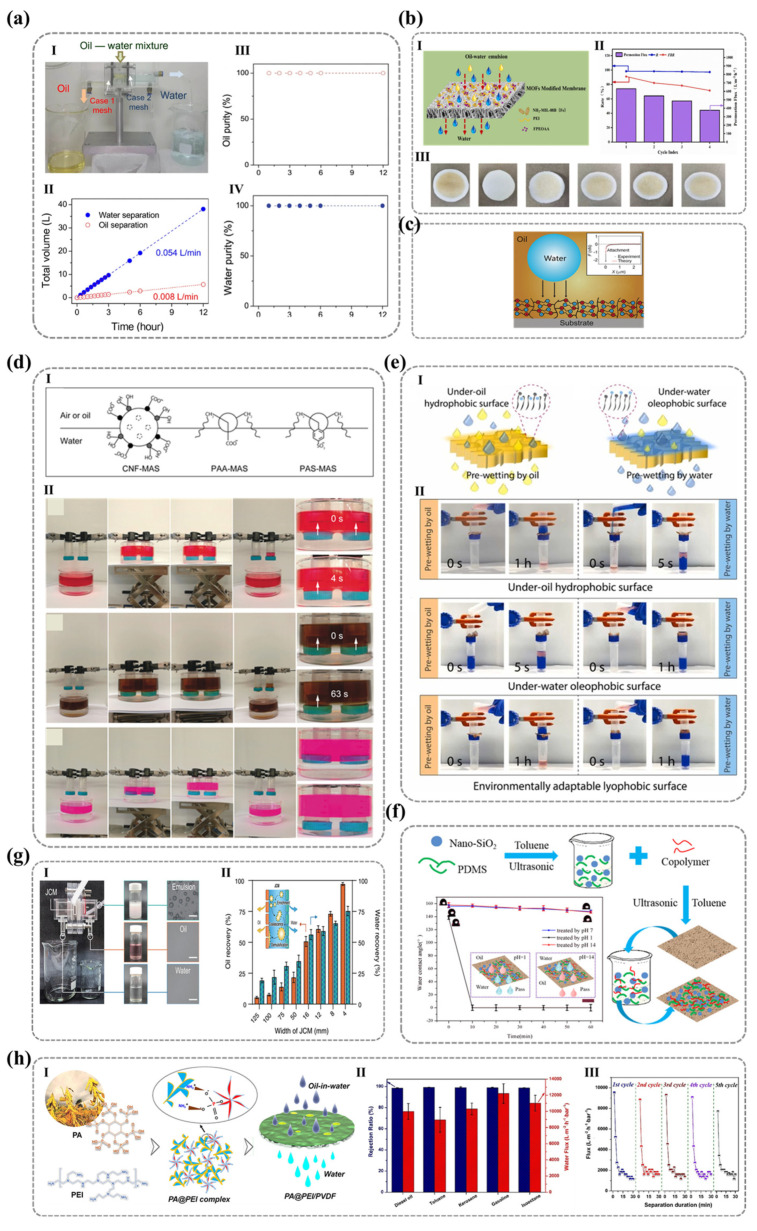
(**a**) Schematic of oil–water separation apparatus with methylene blue-stained water, showing accumulated phase volumes and purity validation over 12 h operation. Ref. [141] Copyright from *Springer Nature*@2021. (**b**) NH_2_-MIL-88B(Fe)/FPEOAA-modified MOF membrane demonstrating pH-stable oil–water separation and cyclic filtration performance. Ref. [142] Copyright from *Elsevier*@2023. (**c**) Long-range hydrophilic attraction mechanisms between water droplets and zwitterionic/cationic/anionic polyelectrolytes in oil medium. Ref. [145] Copyright from *Wiley*@2016. (**d**) Isotropic CNF-coated mesh enabling oil–water separation without pretreatment, contrasted with anisotropic PAA/PSS coatings. Ref. [112] Copyright from *Wiley*@2017. (**e**) Copper-based SAM surfaces achieve selective oil/water filtration through pre-wetting-induced surface reconstruction. Ref. [35] Copyright from *Elsevier*@2024. (**f**) pH-responsive coatings on textiles enabling switchable superhydrophobic/hydrophilic behavior for adaptive oil–water separation. Ref. [146] Copyright from *ASC*@2024. (**g**) Janus-constrained membrane channels (JCM) achieve simultaneous oil/water recovery from emulsions via width-optimized microchannels. Ref. [147] Copyright from *AAAS*@2024. (**h**) PA@PEI/PVDF membrane exhibiting ultrahigh flux and rejection rates for water-in-oil emulsion separation. Ref. [148] Copyright from *Elsevier*@2021.

For instance, Kim et al. (Figure 10a) achieved tunable superhydrophobic/super-oleophilic or super-hydrophilic/underwater super-oleophobic properties by plasma-depositing functional coatings onto stainless-steel meshes. Their system effectively separated oil and water with a remarkable purity of 99.9%, maintaining a continuous water flux of 900 L·m^−2^·h^−1^ and an oil flux of 400 L·m^−2^·h^−1^ [141]. Advancements in ionic polymer materials have further expanded the functional scope beyond basic hydrophilicity. Shen et al. successfully engineered a reusable and acid/alkali-resistant PVDF membrane by employing a condensation reaction between peptide groups in NH_2_-MIL-88B(Fe) and perfluoroalkyl polyoxyethylene acetic acid groups (Figure 10b). This design introduced both hydrophilic amide groups and oleophobic fluorine elements [142]. Additionally, surface charge modulation has been shown to enhance separation efficiency. Studies indicate that adjusting the ζ-potential at mineral interfaces increases electrostatic repulsion between crude oil and the surface, thereby improving recovery rates [143,144]. Chen et al. utilized atomic force microscopy (AFM) to investigate long-range hydrophilic attraction between water droplets and polyelectrolyte surfaces in oil media. Their findings revealed that interactions were strongest with zwitterionic PMAPS surfaces (Figure 10c), attributed to high dipole moments and an ion-hopping mechanism [145].

Recent innovations in molecular design have demonstrated precise control over surface hydrophilicity and charge characteristics. Wang et al. leveraged the anisotropic structure of cellulose nanofibers (CNFs), wherein densely and symmetrically arranged carboxyl and hydroxyl groups form a polar canopy on crystalline nanofiber chains (Figure 10d). This CNF-coated mesh structure efficiently separated a wide range of oils, from viscous engine oil to highly polar butanol, without requiring surface reconstruction or pre-treatment [112]. In comparison, Wang et al. developed an environmentally adaptive binary self-assembled membrane coating by modulating the height disparity between polar and non-polar chains (Figure 10e), enabling selective oil/water filtration through surface reconstruction [35]. Similarly, Du et al. introduced pH-responsive copolymers, demonstrating environmental adaptability. Upon acid treatment, the protonation of PDMAEMA monomers transformed the coating from superhydrophobic to superhydrophilic, allowing for on-demand separation (Figure 10f) [146].

Despite these advancements, significant challenges remain for real-world applications. The stability and durability of hydrophilic polymers under harsh conditions, such as high temperatures, salinity, or organic solvents, continue to be critical concerns, as prolonged exposure often diminishes surface hydrophilicity and separation efficiency. Additionally, complex emulsions, such as surfactant-stabilized systems, require materials with enhanced adaptability. Sustainable manufacturing processes and cost-effective production models are also crucial for large-scale commercialization. Recent research has sought to address these challenges. Guo et al. developed a Janus-constrained membrane (JCM) integrating hydrophilic/oleophobic properties (Figure 10g), optimizing channel width (4 μm) to achieve 97% oil recovery and 75% water recovery from surfactant-stabilized emulsions [147]. Meanwhile, Zeng et al. synthesized an environmentally friendly PA@PEI polyelectrolyte complex using phytate’s strong anionic charge and polyethyleneimine’s cationic density (Figure 10h). This membrane exhibited exceptional underwater superoleophobicity, achieving a 98.5% emulsion rejection rate and an ultra-high water flux of 12,203.6 L·m^−2^·h^−1^·bar^−1^ [148].

These breakthroughs underscore the transformative potential of surface-functionalized membranes in oil–water separation, paving the way for more efficient, scalable, and sustainable wastewater treatment technologies.

### 3.2. Hydrophilic Anti-Fogging ($f_{p}^{M}>0.65$) and Cryo-Tolerant Anti-Icing Interfaces ($f_{p}^{M}>0.85$)

In high-humidity environments, temperature fluctuations induce the heterogeneous condensation of water vapor, leading to the formation of fog (micron-sized liquid droplets) or ice crystals (solid deposits) on critical surfaces such as windshields, protective eyewear, and optical devices. These phenomena severely compromise operational functionality and safety across various industries—obscuring visibility in transportation, altering the aerodynamic profile of aircraft wings, and precipitating structural failures in power grids due to ice accumulation on transmission lines [149]. Conventional mitigation strategies, including chemical de-icing agents and mechanical scraping, are often inefficient, environmentally detrimental, and economically unsustainable [150,151].

**Figure 11:**
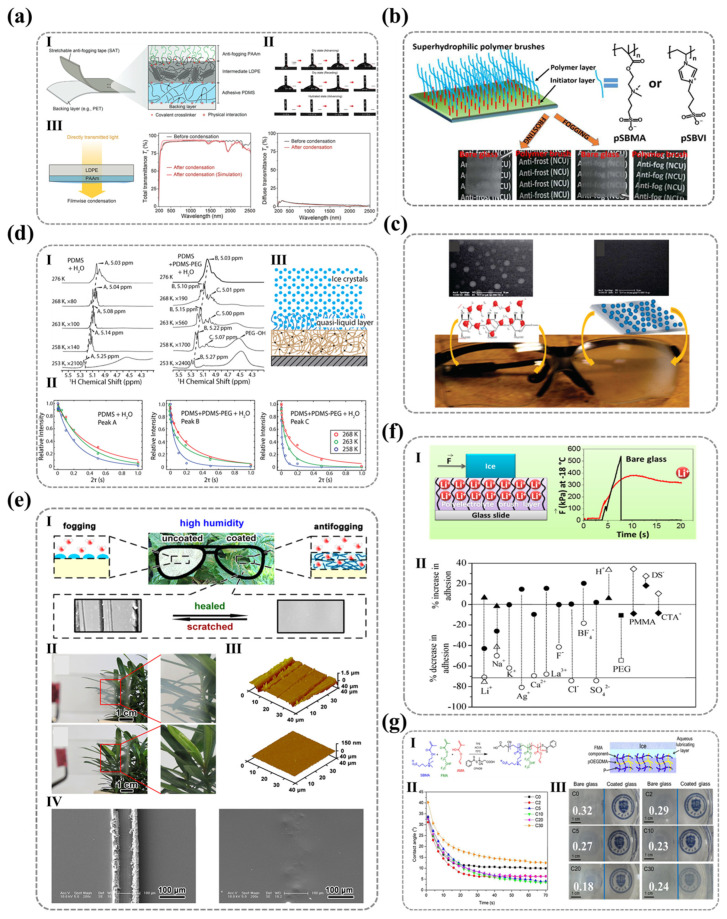
(**a**) Stretchable anti-fog tape (SAT) demonstrating humidity-responsive wettability and optical stability under condensation. Ref. [152] Copyright from *Wiley*@2021. (**b**) Ultra−hydrophilic zwitterionic polymer brushes (pSBMA/pSBVI) exhibiting frost−resistant performance compared to bare glass. Ref. [155] Copyright from *RSC*@2016. (**c**) Anti-fog mechanisms of polysaccharide films via water film formation versus droplet nucleation on conventional polyelectrolytes. Ref. [153] Copyright from *ACS*@2010. (**d**) Non−freezing quasi-liquid layer (QLL) formation on PDMS−PEG surfaces revealed by NMR relaxometry and ice adhesion reduction. Ref. [154] Copyright from *ACS*@2017. (**e**) Self−healing anti−freeze/anti-fog coatings regenerating scratched surfaces under ambient humidity. Ref. [157] Copyright from *ACS*@2015. (**f**) Polymer brush surfaces with Li^+^ counterions modulating ice adhesion strength across temperatures. Ref. [156] Copyright from *ACS*@2014. (**g**) RAFT−synthesized zwitterionic/OEGDMA copolymer coatings balancing anti−fog durability and hydrophilicity. Ref. [158] Copyright from the *Elsevier*@2018.

To circumvent these limitations, engineering surfaces with superior wettability have emerged as a promising approach to suppress fog formation by enabling complete droplet spreading, thereby preventing light refraction and optical distortion. The strategic incorporation of highly polar functional groups (e.g., –OH and –COOH) onto solid surfaces enhances hydration capacity, facilitating persistent anti-fogging performance. Lin et al. exemplified this principle through the development of a stretchable anti-fog tape (SAT) composed of polyacrylamide (PAAm), which retained over 90% optical transmittance even under 200% strain by promoting the formation of a uniform hydration layer (Figure 11a) [152]. Complementary evidence from Nuraje et al. demonstrated via environmental scanning electron microscopy (SEM) that a polysaccharide-based coating established a robust hydrogen-bonding network with water molecules, preventing the nucleation of fog droplets [153], as shown in Figure 11c.

In contrast, ice formation entails the direct deposition of water vapor into the solid phase through condensation, necessitating the inhibition of ice nucleation and the reduction in ice adhesion forces to facilitate rapid ice detachment from surfaces. Chen et al. reported that at −15 °C, polyethylene glycol (PEG) surfaces increased the viscosity of interfacial water by an order of magnitude, effectively retarding ice nucleation while preserving a lubricating unfrozen quasi-liquid layer at 258 K (Figure 11d) [154]. In a parallel study, Ezzat and Huang engineered a dual-charged ionic polymer brush (pSBMA/pSBVI) that exhibited exceptional anti-frost properties at −20 °C, underscoring the pivotal role of ionic functional groups in stabilizing interfacial water structures (Figure 11b) [155]. Furthermore, Chernyy et al. tailored polyelectrolyte brushes using Li^+^ counterions, thereby enhancing the hydration of the quasi-liquid layer (QLL) and achieving a 70% reduction in ice adhesion at −10 °C (Figure 11f) [156].

One inherent limitation of hydrophilic anti-icing coatings is their susceptibility to degradation over time. To extend their functional lifespan, Wang et al. devised a self-healing multilayer material (PEI/HA-PAA) × 50, leveraging reversible electrostatic and hydrogen-bonding interactions to facilitate autonomous scratch repair (55 μm closure distance) upon water exposure, thus preserving both optical transparency and anti-icing efficacy (Figure 10e) [157]. Additionally, conventional hydrophilic coatings often exhibit long-term instability due to excessive water absorption, leading to compromised structural integrity. Addressing this challenge, Tao et al. synthesized semi-interpenetrating polymer networks (SIPNs) via reversible addition–fragmentation chain transfer (RAFT) polymerization, incorporating a precisely tuned balance of hydrophobic fluorinated monomers (FMA/AMA) and dual-charged ionic polymer brushes (SBMA). This strategic modulation of the hydrophilic–hydrophobic equilibrium ensured durable anti-fogging and anti-icing performance over prolonged usage (Figure 11g) [158].

These advances underscore the imperative of molecular-level optimization in achieving a synergistic balance between interfacial hydration, durability, and environmental resilience, thereby facilitating next-generation anti-fogging and anti-icing materials with superior adaptability and performance.

### 3.3. Hydration-Barrier Strategies Against Paraffin Fouling in Petroleum Systems (Anti-Waxing ($f_{p}^{M}>0.85$))

In the petroleum sector, wax deposition predominantly arises from the crystallization and precipitation of long-chain saturated alkanes (C_18_–C_60_) under sub-ambient conditions. Once adhered to pipeline interiors, these wax layers drastically diminish the effective transport cross-section, exacerbating flow resistance and amplifying the energy demand for pumping operations. Moreover, their intrinsically low dielectric constant (ε ≈ 2.3) impairs the dissipation of electrostatic charges engendered by friction between flowing crude oil and solidified wax deposits. Consequently, localized charge accumulation poses a formidable hazard, potentially triggering electrostatic discharges or even catastrophic explosions, thereby underscoring the criticality of mitigating wax accretion to ensure the integrity and reliability of petroleum infrastructure.

**Figure 12:**
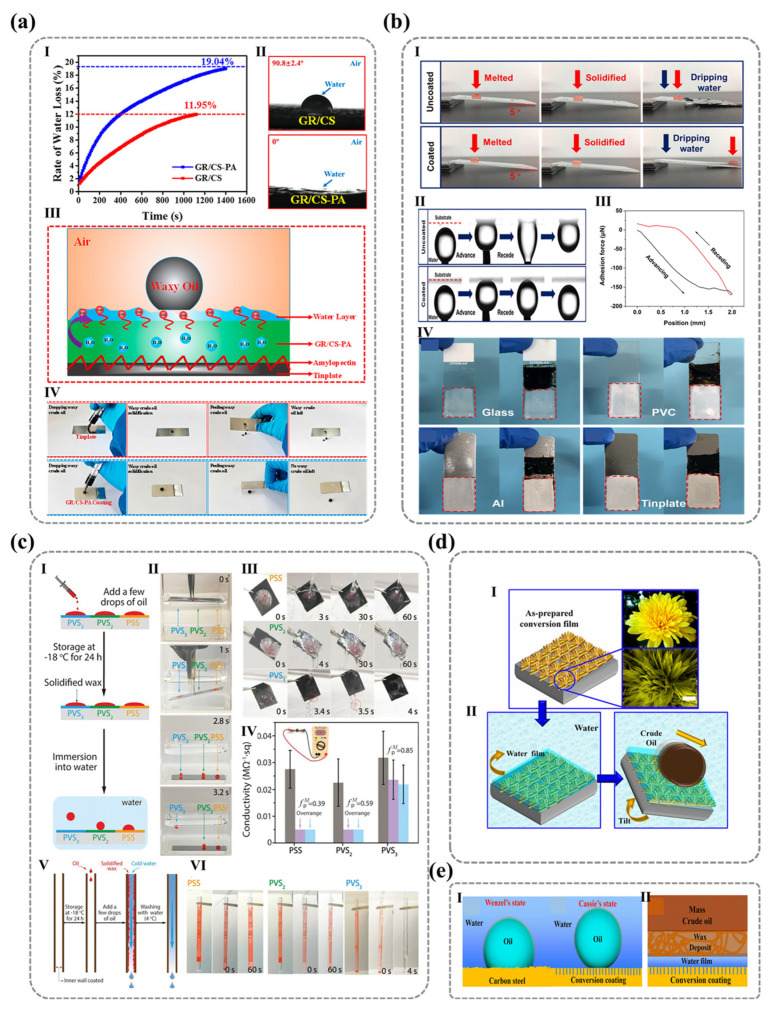
(**a**) Thermal stability and anti-wax performance of GR/CS−PA coatings. Ref. [164] Copyright from *ACS*@2018. (**b**) PVA/zeolite−15 coated surfaces exhibiting wax resistance, low oil adhesion, and substrate-independent self−cleaning capabilities. Ref. [165] Copyright from *Elsevier*@2019. (**c**) Sulfonated coatings (PSS−CSs/PVS−CSs) enabling rapid cold−water wax removal. Ref. [138] Copyright from *Wiley*@2023. (**d**) Biomimetic chrysanthemum−like conversion coating with underwater superoleophobicity for wax deposition inhibition. Ref. [167] Copyright from *ACS*@2013. (**e**) Hydrophilic conversion coating preventing oil adhesion and enabling self−cleaning in water−containing crude oil systems. Ref. [166] Copyright from *Elsevier*@2013.

Conventional remediation techniques—ranging from mechanical scraping [159], electrical heating [160], and ultrasonic agitation [161] to microbial degradation [162] and chemical wax inhibitors [163]—frequently suffer from inefficiencies, exorbitant energy consumption, or operational impracticalities, necessitating the development of more efficacious and sustainable solutions. Hydrophilic membranes have emerged as a paradigm-shifting approach, leveraging their intrinsic hydrophilic–oleophobic properties to mitigate wax adhesion, hinder accumulation, and provide environmentally benign alternatives. Recent advances in surface engineering have propelled hydrophilic coatings to the forefront of anti-wax strategies due to their unique physicochemical attributes. By augmenting surface water affinity, these coatings facilitate the formation of persistent, defect-free aqueous films that act as dual-function barriers—physically impeding wax deposition while concurrently dissipating electrostatic charges. For instance, Wang et al. pioneered a chitosan-phytic acid (CS-PA)/glutinous rice (GR) composite coating, wherein the ionic crosslinked chitosan-phytic acid network establishes a robust water-retentive layer, while GR serves as a binding matrix, yielding a water contact angle below 10° and sustaining over 95% water retention (Figure 12a). Under simulated deep-sea low-temperature conditions, this system achieved an impressive 80% reduction in wax deposition [164].

Similarly, Li et al. developed a bioinspired PVA/zeolite composite coating (Figure 12b), wherein the synergistic interplay between polyvinyl alcohol (PVA) and zeolite’s hierarchical micro-nano architecture confers underwater superoleophobicity (oil contact angle > 160°) and self-cleaning functionality, offering an effective strategy for maintaining oil-contaminated pipelines [165]. In the realm of chemical conversion coatings, Wang et al. employed phosphoric acid treatments to synthesize flower-like polyphosphate coatings exhibiting remarkable superoleophobicity (oil contact angle: 162°, sliding angle: 7°), thereby curtailing wax deposition to a mere 2.9 mg/cm^2^—an 80% reduction compared to untreated surfaces [166]). Inspired by the microstructures of fish scales and shark skin, another study introduced a biomimetic iron phosphate coating fabricated via in situ AC deposition within a mere four-hour timeframe. The resultant fractal architecture not only effectively suppresses wax accretion but also bolsters corrosion resistance (corrosion rate: 0.03 mm/year), heralding a promising avenue for high-wax oilfield pipeline applications.). Expanding upon this concept, Wang et al. further refined lithium iron phosphate conversion films via AC deposition (Figure 12d), achieving wax-repellent and self-cleaning properties in oil-in-water emulsions through a streamlined single-step process completed in under four hours [167].

To elucidate the molecular-scale correlation between hydrophilicity and wax resistance, Wang’s team strategically optimized the surface density of sulfonate functional groups (targeting $f_{p}^{M}\approx$ 0.9) to engender densely packed hydration layers. This precisely engineered interface facilitates the complete removal of wax deposits from capillary structures within an astonishingly brief four-second cold-water flush, drastically curtailing maintenance expenditures for low-flow pipelines (Figure 12c) [138]. These advanced hydrophilic coatings signify a groundbreaking trajectory for future research, seamlessly integrating operational efficacy with scalable manufacturability.

## 4. Conclusions and Outlook

### 4.1. Conclusions

In this review, we have underscored the critical role of molecular-level material design in modulating surface wettability. We have provided a comprehensive analysis of methodologies for engineering nanoscale smooth hydrophilic polymer surfaces, encompassing not only fabrication techniques but also the fundamental molecular determinants governing their behavior. Furthermore, we have systematically evaluated applications such as oil–water separation, anti-fogging, anti-icing, and anti-waxing based on varying hydrophilicity requirements by considering the collective effects of both polar and nonpolar moieties on surfaces. Through illustrative case studies, we have further demonstrated how molecular design strategies can be tailored to meet specialized functional demands.

**Figure 13:**
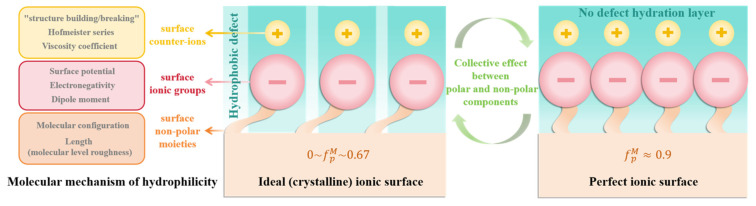
Schematic of the molecular mechanism of ion-type surface hydrophilicity.

The molecular mechanisms underlying the hydrophilicity of charged polymer surfaces can be elegantly distilled as follows (Figure 13): the hydrophilic nature of these surfaces is predominantly dictated by those ionic polar groups. Specifically, the type, density, and counterion species of ionic terminal groups collectively govern surface hydration efficiency. Moreover, the geometric configuration of adjacent non-polar groups modulates the persistence of surface hydration, ensuring stable hydrophilicity across diverse environmental conditions. Thus, achieving optimal surface hydration necessitates a holistic consideration of the interplay between polar and non-polar moieties, ensuring that the hydration of polar groups is sufficiently robust to mask underlying hydrophobic domains.

### 4.2. Outlook

While significant progress has been made in elucidating the molecular mechanisms of hydrophilicity in ionic polymer surfaces, numerous intricate aspects remain ripe for further exploration. Several promising avenues for future investigation include:

**A.**  **Surface Reconstruction Induced by Adjacent Non-Polar Groups:**

**Figure 14:**
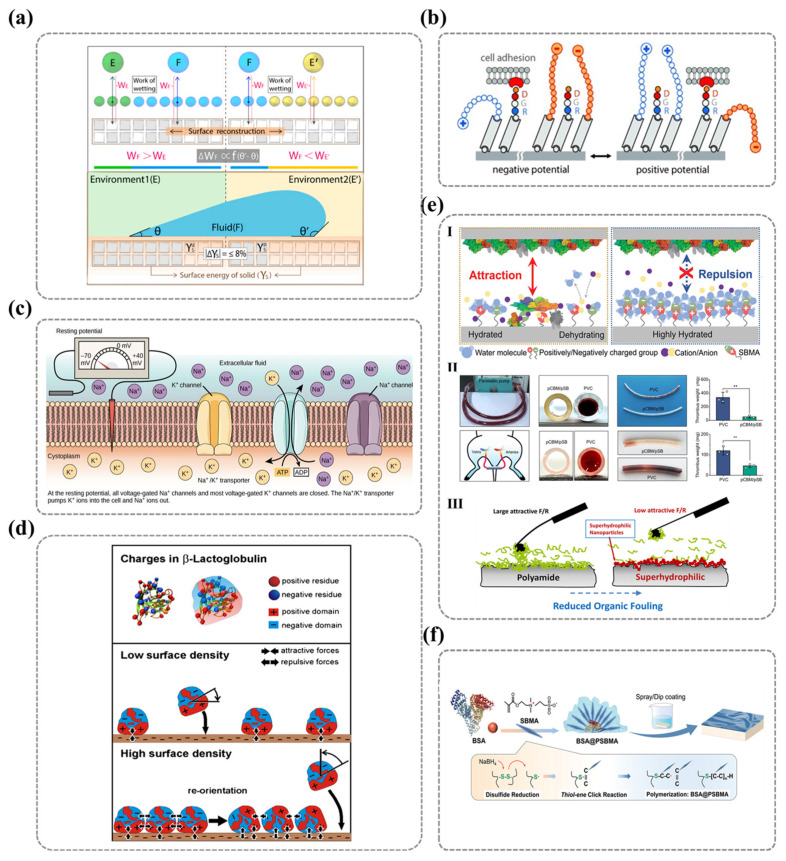
(**a**) Schematic showing the competition between macroscopic wettability and adhesion work of the wetting phase ($W^{f}$) and environmental wettability work ($W^{e}$). Ref. [111] Copyright from *Elsevier*@2023. (**b**) Schematic of an intelligent surface with charged functional groups on the distal end and molecules terminating with RGD peptides for cell adhesion, which can dynamically switch by applying +300 or −300 mV to allow or prevent cell proximity to the peptide. Ref. [169] Copyright from *Wiley*@2012. (**c**) Schematic illustrating the distinct selectivity of sodium (Na^+^) and chloride (Cl^−^) ions in ion channel transport within cells, despite their comparable $B_{\eta }$ values and similar positions in the Hofmeister series, highlighting their role in the differential distribution of sodium and potassium ions in nerve and muscle cells and the generation of potential differences (downloaded from: Bioelectricity—Human Physiology (uoguelph.ca)). (**d**) Schematic showing protein adsorption on surfaces with low and high charge densities. Ref. [171] Copyright from *Elsevier*@2011. (**e**) I. Schematic of anti-fouling/scaling via hydration layers. Ref. [173] Copyright from *Wiley*@2022. II. Antithrombotic results in vitro and ex vivo from a rat model with pCBM/pSB hydrogel coating and bare PVC tubing. Ref. [174] Copyright from *Springer Nature*@2022. III. Schematic of anti-organic contaminant FO/RO permeation membrane achieved by modifying superhydrophilic nanoparticles. Ref. [175] Copyright from *ACS*@2012. (**f**) Schematic of hydrophilic coating surface preparation by thiol-ene click chemistry to synthesize BSA@PSBMA. Ref. [173] Copyright from the *Wiley*@2022.

The extent and likelihood of surface reconstruction driven by adjacent non-polar groups can be inferred from discrepancies between the ideal ($\theta_{l/e}^{cal}$) and experimentally observed ($\theta_{l/e}^{exp}$) contact angles ($\delta_{l/e}$), as well as disparities in the polar ($\gamma^{p}$) and non-polar ($\gamma^{d}$) components of surface energy. Existing thermodynamic indicators (e.g., deviation in adhesion W Figure 14a) [111] have been developed to assess molecular-scale reconstruction in non-ionic polar surfaces. However, for charged polar surfaces, particularly in the context of dynamic reconstruction processes, a self-consistent, quantitative thermodynamic framework related to wettability remains elusive.

**B.**  **Molecular-Scale Roughness of Layer-by-Layer (LBL) Self-Assembled Multilayers:**

Ionic group-functionalized surfaces constructed via LBL assembly—such as those composed of strong polyelectrolytes ((PDDA/PSS)_n_)—offer a unique platform for investigating counterions and charged moieties at the molecular scale. However, in practical applications, molecular-scale roughness in LBL-assembled surfaces can escalate due to the intrinsic properties of membrane materials or the number of assembly layers [168]. In such cases, the surface composition resembles that of self-assembled monolayer (SAM) films with multiple polar components. For instance, as shown in Figure 14b, Gooding et al. demonstrated that in SAMs terminated with binary polar end groups, the intrinsic anti-cell adhesion properties were lost when charged end groups (EG6) underwent inward surface reconstruction due to surface potential shifts [169]. Yet, the wettability and anti-fouling performance of LBL multilayers with molecular-scale roughness remains an underexplored frontier.

**C.**  **Quantitative Characterization of Counterion Hydration Capacity** **:**

Although the viscosity coefficient $B_{\eta }$ offers preliminary qualitative insights into whether ions “structure” or “disrupt” water networks, the wettability differences among ions with identical $B_{\eta }$ values remain quantitatively underexplored. For instance, despite sodium (Na^+^) and chloride (Cl^−^) ions exhibiting comparable $B_{\eta }$ values and similar positions in the Hofmeister series, they demonstrate markedly different selectivity in ion channel transport within cells (Figure 14c), contributing to the distinct distribution of sodium and potassium ions in nerve and muscle cells and the generation of potential differences [170].

**D.**  **Enhancing Surface Hydration and Mitigating Hydrophobic Defect Exposure** **:**

Recent studies have introduced collective molecular parameters $f_{p}^{\theta }$ and $f_{p}^{M}$ to classify surface hydrophilicity based on the packing density of polar moieties. When polar groups achieve the maximum density of two-dimensional random packing spheres (approximately 0.67), the surface hydration capacity meets basic hydrophilic requirements. At the theoretical maximum density of two-dimensional close-packed spheres (approximately 0.9), hydrophobic regions are effectively masked, enabling superhydrophilicity for highly demanding applications. However, these studies primarily focus on interactions between polar and non-polar groups. When surfaces incorporate multiple polar and non-polar functionalities or feature intricate configurations, their synergistic effects on wettability warrant further in-depth investigation.

**E.**  **Quantitative Self-Cleaning Applications in Air and Underwater** **:**

Current quantitative assessments of hydrophilic self-cleaning applications primarily focus on the environment of air. However, in underwater anti-fouling applications, while superior wettability is crucial, the dissociation and charge variations of certain contaminants complicate the mechanisms governing hydrophilic ionic surfaces. For instance, medical instruments, ship hulls, and reverse-osmosis membranes frequently encounter proteins bearing both positive and negative charges [171]. This necessitates the consideration of not only surface hydrophilicity but also electrostatic interactions between charged surfaces (Figure 14d). Consequently, molecular designs must meet more stringent requirements. Beyond highly hydrophilic zwitterions (Figure 14e), researchers have discovered that surfaces functionalized with natural proteins (e.g., bovine serum albumin, BSA) exhibit exceptional anti-protein adhesion properties (Figure 14e) [172,173]. Thus, biomimetic surface designs inspired by natural proteins may represent a promising strategy for next-generation anti-fouling materials.

**F.**  **Challenges in Hydrophilic Material Stability and Environmental Responsiveness** **:**

With the continuous evolution of industrial and biomedical technologies, the demand for advanced hydrophilic materials is surging. However, maintaining stable hydrophilicity under extreme conditions—such as high temperatures, high pressures, and exposure to strong acids or bases—remains a formidable challenge. Additionally, certain applications require materials to be biodegradable and environmentally responsive. As sustainable development gains momentum, the design of eco-friendly materials has become an imperative research focus. Developing hydrophilic polymers that exhibit both long-term stability and controlled degradation presents a novel challenge in ionic polymer material science.

In conclusion, the exploration of molecular mechanisms governing nanoscale smooth hydrophilic ionic polymer surfaces remains a dynamic and rapidly evolving domain, replete with both challenges and opportunities. With the continuous advancement of molecular-level characterization techniques—including X-ray and neutron diffraction, sum-frequency generation (SFG) spectroscopy, nuclear magnetic resonance (NMR) spectroscopy, and molecular dynamics (MD) simulations—we are confident that future research will progressively unravel the intricate mysteries of ionic surface hydrophilicity, ultimately providing invaluable insights for the future molecular design and real-world application of next-generation hydrophilic materials.
